# The wide expansion of hepatitis delta virus-like ribozymes throughout trypanosomatid genomes is linked to the spreading of L1*Tc*/*ingi* clade mobile elements

**DOI:** 10.1186/1471-2164-15-340

**Published:** 2014-05-06

**Authors:** Francisco José Sánchez-Luque, Manuel Carlos López, Patricia Eugenia Carreira, Carlos Alonso, María Carmen Thomas

**Affiliations:** Instituto de Parasitología y Biomedicina “López-Neyra”, CSIC, Parque Tecnológico de Ciencias de la Salud, Av. del Conocimiento s/n, 18016 Granada, Spain; Centro de Biología Molecular “Severo Ochoa”, CSIC-UAM, Campus Universidad Autónoma de Madrid, C/Nicolás Cabrera n°1, 28049 Madrid, Spain

**Keywords:** Retrotransposon, LINE, SINE, HDV-like ribozyme, Pr77, *Trypanosoma*, *Leishmania*, L1*Tc*, SIDER, *ingi*

## Abstract

**Background:**

Hepatitis Delta Virus (HDV)-like ribozymes have recently been found in many mobile elements in which they take part in a mechanism that releases intermediate RNAs from cellular co-transcripts. L1*Tc* in *Trypanosoma cruzi* is one of the elements in which such a ribozyme is located. It lies in the so-called Pr77-hallmark, a conserved region shared by retrotransposons belonging to the trypanosomatid L1*Tc*/*ingi* clade. The wide distribution of the Pr77-hallmark detected in trypanosomatid retrotransposons renders the potential catalytic activity of these elements worthy of study: their distribution might contribute to host genetic regulation at the mRNA level. Indeed, in *Leishmania spp*, the pervasive presence of these HDV-like ribozyme-containing mobile elements in certain 3′-untranslated regions of protein-coding genes has been linked to mRNA downregulation.

**Results:**

Intensive screening of publicly available trypanosomatid genomes, combined with manual folding analyses, allowed the isolation of putatively Pr77-hallmarks with HDV-like ribozyme activity. This work describes the conservation of an HDV-like ribozyme structure in the Pr77 sequence of retrotransposons in a wide range of trypanosomatids, the catalytic function of which is maintained in the majority.

These results are consistent with the previously suggested common phylogenetic origin of the elements that belong to this clade, although in some cases loss of functionality appears to have occurred and/or perhaps molecular domestication by the host.

**Conclusions:**

These HDV-like ribozymes are widely distributed within retrotransposons across trypanosomatid genomes. This type of ribozyme was once thought to be rare in nature, but in fact it would seem to be abundant in trypanosomatid transcripts. It can even form part of the pool of mRNA 3′-untranslated regions, particularly in *Leishmania spp*. Its putative regulatory role in host genetic expression is discussed.

**Electronic supplementary material:**

The online version of this article (doi:10.1186/1471-2164-15-340) contains supplementary material, which is available to authorized users.

## Background

Retrotransposons are mobile DNA elements that mobilise via a copy-paste mechanism using an intermediate RNA to propagate new copies throughout the host genome. As a consequence of their activity, these repeated sequences can make up large proportions of eukaryote genomes. Non-long terminal repeat (non-LTR) retrotransposons mobilise using a target-primed reverse transcription (TPRT) mechanism involving the use of the 3′ hydroxyl group at a DNA break to prime the reverse transcription of their RNAs [1]. As a consequence of this TPRT mechanism, short direct target site duplications (TSDs) flank the newly inserted copies.

**Figure 1:**
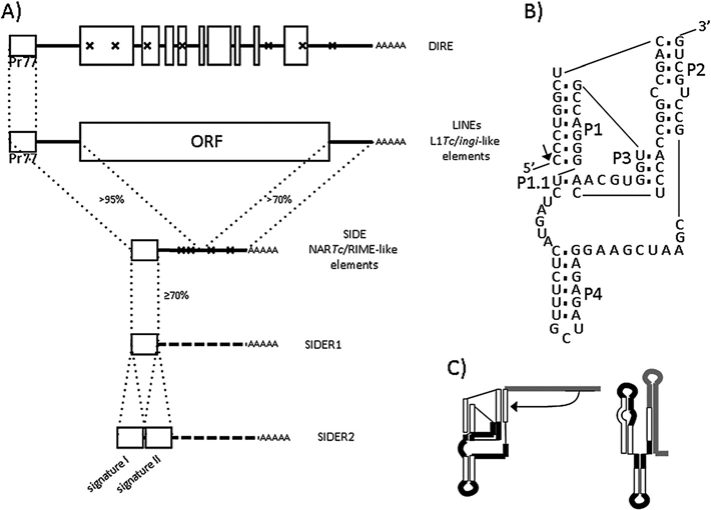
**HDV-like ribozymes in trypanosomatid mobile elements.** The structure of L1*Tc*/*ingi* clade mobile elements is shown in **(A)**. The wide white boxes indicate ORFs or degenerated ORFs (mutations are represented as crosses inside white boxes). The narrow white boxes located at the 5'-ends of all elements indicate the common and conserved Pr77-hallmark and the percentage similarity at the nucleotide level. Dotted lines mark out the conserved regions in long and short elements. LINEs (Long Interspersed Nucleotide Elements) such as L1*Tc* and *ingi* and the DIRE or L1*Tc*/*ingi* degenerated versions, are indicated at the top of panel **A**. NAR*Tc* and RIME are truncated versions of L1*Tc* and *ingi* elements and are shown at the bottom of panel **A**; these elements are also known as SIDE (Short Internally Deleted Elements) and short interspersed degenerate elements bearing one (SIDER1) or two (SIDER2) Pr77-hallmarks, referred as signature I and II, The proposed folding for L1*Tc*Rz [25], the HDV-like ribozyme previously described in L1*Tc* from *Trypanosoma cruzi*, is shown in **(B)**. The arrow indicates the cleavage point and relevant structural helixes (P1, P2 and P4) and pseudoknots (P1.1 and P3). The switch from L1*Tc*Rz to a three-helix structure proposed for after L1*Tc* downstream region transcription [25] is shown in **(C)**. The white boxes in **C**, indicate the structural components of the HDV-like ribozyme helixes. The black lines indicate single stranded regions. The grey lines indicate the attenuator downstream region.

Non-LTR retrotransposons can be classified into long and short interspersed nuclear elements (LINE and SINE respectively [2]; Figure 1A). LINEs are mobilised in an autonomous fashion by the retrotransposition machinery they encode. They contain one or two open reading frames (ORF) and are transcribed and translated by the cellular machinery. SINEs, which have no ORF and code for no protein, are mobilised *in trans* by the LINE-encoded enzymatic machinery. SINEs are either products of LINE ORF deletion, with preservation of the LINE sequence 5′- and 3′-ends (also referred to as short, internally deleted elements or SIDEs [3]; Figure 1A) or chimeras of cellular, viral or other transposable element RNAs (e.g., Alu, SVA, 5SrRNA- and tRNA-chimeric elements) that carry an internal promoter in the 5′-end region [4–6].

Trypanosomatid genomes are highly colonised by repeats of mobile elements belonging to the L1*Tc*/*ingi* clade (also known as the *ingi* clade); these elements are the best represented retrotransposons in these organisms [7, 8]. L1*Tc* and *ingi* are LINEs found in the genomes of *Trypanosoma cruzi*[9] and *Trypanosoma brucei*[10], the agents responsible for American and African human trypanosomiasis (Chagas’ disease and sleeping sickness) respectively. L1Tc is a potentially functional autonomous retrotransposon that encodes its own retrotransposition machinery, which involves apurinic/apyrimidinic endonuclease, reverse transcriptase, RNase H, and nucleic acid chaperone activities [11–14]. NAR*Tc* (a non-autonomous retrotransposon [15]) and RIME (ribosomal inserted mobile element [16]) are truncated versions of the L1*Tc* and *ingi* elements respectively. *Trypanosoma* and *Leishmania spp* genomes also contain highly degenerate elements of long and short length related to retrotransposons of the L1*Tc*/*ingi* clade named DIREs (Degenerated *ingi*-Related Elements) [17] and SIDERs (Short Internally Degenerated Retroposons) respectively (Figure 1A). These elements are unable to mobilise by themselves.

While the transcription of several non-LTR retrotransposons is driven by an internal promoter encoded at their 5′-end [18–20], others are transcribed starting at host promoters located upstream of the element insertion site [21, 22]. Recently, a Hepatitis Delta Virus (HDV)-like ribozyme has been described as the device responsible for the release of retrotransposon RNAs in the insect R2 (R2Rz) and the *T. cruzi* L1*Tc* (L1*Tc*Rz) elements [20–26]. Catalytic cleavage occurs just upstream of the ribozyme domain. Other members of the HDV-like ribozyme family have been described in the human genome [27] as well as in insects, plants and fish, in which they have been shown to be functional [28].

L1*Tc*/NAR*Tc* and *ingi*/RIME are the most abundant repeat elements in the *T. cruzi* and *T. brucei* genomes. The 77 nt-long conserved sequence at their 5′-ends, known as the Pr77-hallmark, has been shown to work as an internal promoter (at the DNA level) and as an HDV-like ribozyme at the RNA level (Figure 1B) ([25, 29, 30], Carreira P, López MC *et al.* manuscript in preparation). This Pr77-hallmark is also conserved in other LINEs and SINEs residing in the genome of *Trypanosoma vivax, Trypanosoma congolense* and *T. brucei*, as well as in DIREs of trypanosomatids and SIDERs in the genomes of *Leishmania spp*[7]*.*

Typanosomatid genomes are organised as large clusters of gene tandems constitutively transcribed by RNA polymerase II launched from regions between the clusters (strand switch regions or SSR [31, 32]). The great accumulation of mobile elements within the SSRs suggests that Pr77-hallmark promoter activity is involved in the epigenetic recruitment of RNA polymerase II [29].

Mature mRNAs are generated by *trans*-splicing of a short-capped RNA, called a spliced leader, which is transcribed from a widely repeated genetic unit via a unique polymerase II external promoter. *Trans-*splicing is coupled to the polyadenylation of the preceding pre-mRNA unit in the polycistronic RNA. The regulation of transcription is mainly attributed to post-transcriptional processes related to the stability of mRNAs. It has been reported that unstable *Leishmania* mRNAs harbouring a SIDER2 retrotransposon in the 3′-untranslated region (a group of SIDERs which bear two in-tandem Pr77-nt hallmarks at their 5′end, see diagram in Figure 1A) are degraded by site-specific endonucleolytic cleavage that takes place at the 5′-end of the second 77-nt signature (referred to as signature II) [33–35].

The Pr77-hallmark RNA from L1*Tc* has an HDV-like ribozyme secondary structure [25]. The RNA region located immediately downstream of Pr77 in L1*Tc*, but not in NAR*Tc*, attenuates the *in vitro* co-transcriptional activity of the ribozyme, probably via rapid T7 RNA polymerase transcription. Most likely, *in vivo* this region induces a structural switch of the 5′-UTR after ribozyme cleavage (Figure 1C). *In vitro*, the structure of the entire 5′-UTR is recognised as a tRNA-like structure in L1*Tc* by *Escherichia coli* RNase P M1 RNA [25], suggesting that the 5′ UTR switches into a structure involved in translation [36].

This paper reports that HDV-like ribozymes are also present in most of the L1*Tc*/*ingi* clade retrotransposons, which are ubiquitous in trypanosomatids. This suggests that, as observed for the R2 retrotransposon clade in insects [26], they are the result of the phylogenetic inheritance of a common mobile element ancestor (not a product of horizontal transfer). They therefore likely have a common role in the genetic regulation of their hosts. Pr77-hallmark promoter and ribozyme activities may play important roles in trypanosomatid genetic regulation.

## Results and discussion

### Identification of putatively active HDV-like ribozyme candidates related to the L1Tc/ingi clade in trypanosomatids

**Figure 2:**
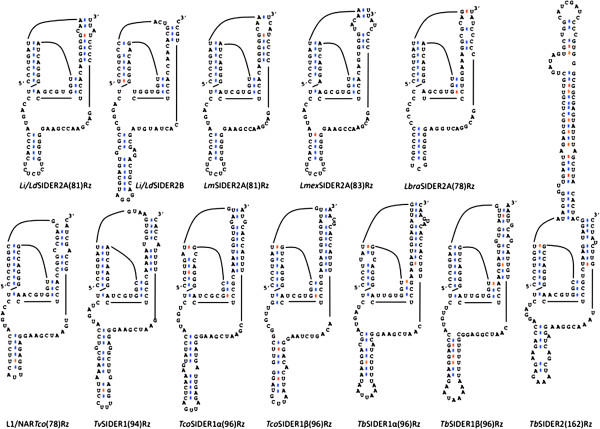
**Selected ribozymes putatively active based on their folding.** All the screened sequences (see Methods) were manually folded and those compatible with previously described ribozyme requirements or with minimum alterations were selected for study. The *Leishmania spp* sequences correspond to those identified in the SIDER elements of the insertion referred to in the text as insertion 1. *Li/Ld*SIDER2B is included in the figure, even though it was not included in the study, to show that the first signature in *Leishmania* SIDER2 elements is a candidate for ribozyme activity. Helix P3 and *pseudo*knot P1.1 are not correctly folded in the *Li/Ld*SIDER2B signature. Watson-Crick base pairs are depicted in blue and wobble ones in red.

A bioinformatic BLAST search of the Eukaryotic Pathogen Resources Database (EuPathDB) was used to search for Pr77 homologues in mobile elements of different trypanosomatid genomes (*T. brucei*, *T. congolense*, *T. vivax*, *Leishmania major*, *Leishmania donovani*, *Leishmania infantum*, *Leishmania tarentolae*, *Leishmania braziliensis*, *Leishmania panamensis* and *Leishmania mexicana*). The Pr77-hallmark consensus [37] was used as an initial query in screenings (see Methods for details). The putative HDV-like ribozyme structure of each Pr77 signature was manually explored. The sequence of the identified mobile elements that adopted any folding compatible with an HDV-like ribozyme and the correspondant genomic annotations are given in Additional file 1: Figure S1. For the *in vitro* analysis, those that better fitted the previously described folding requirements of the L1*Tc* or R2 retrotransposons (Figure 2) were chosen.

SIDER elements were found in all the analysed trypanosomatid species. The members of the SIDER1 subgroup contained a single Pr77 signature located at their 5′-end, while those of the SIDER2 subgroup contained two signatures (called SIDER2A and SIDER2B; or signature I and II depending on the literature examined [35, 38]). Elements of the SIDER2 subgroup were identified in *L. infantum, L. donovani, L. major, L. mexicana* and *L. braziliensis*. The first signature of the SIDER2 elements seems to fit the HDV-like ribozyme folding of L1*Tc*Rz (Figure 2 and Additional file 1: Figure S1). SIDER copies from *Leishmania spp* located in two different positions of the genome were selected for phylogenetic analyses (Additional file 1). One of them (referred to as insertion 1) is located at the 3′-UTR of the gene coding for a putative mitochondrial DEAD box protein. The other (referred to as insertion 2) is located at the 3′-UTR of the gene coding for a hypothetical protein of unknown function which is conserved and syntenic in all publicly available *Leishmania* genomes (upstream gene localisers LmjF.29.2290; LinJ.29.2400/LdBPK_282220.1, LmxM.08_29.2290; LtaP29.2440 and LbrM.29.2260). Only the ribozymes belonging to the insertion 1 SIDERs in the different *Leishmania spp* were assayed for the analysis of ribozyme function (Figure 2 and Additional file 1: Figure S1).

A single Pr77 signature was found in *T. congolense* L1*Tco* and NAR*Tco* (L1/NAR*Tco*(78)Rz), and in *T. vivax* SIDER1 (*Tv*SIDER1(94)Rz), with folding similar to that shown by the L1*Tc* ribozyme. In addition, two in-tandem Pr77-hallmarks were found in *T. brucei* SIDER2 (*Tb*SIDER2(126)Rz, Figure 2 and Additional file 1: Table S1) showing a hybrid folding of ribozymes L1*Tc* and R2 [23, 25]. No SIDER1 signature was found, either in *T. brucei* or *T. congolense* that fitted any folding compatible with an HDV-like ribozyme. Thus, for *in vitro* transcription analysis, two elements were chosen from each species that showed minor structural disruptions (called *Tco-* and *Tb-* SIDER1α(96)Rz and SIDER1β(96)Rz; Figure 2, Additional file 1: Figure S1, Figure S2 and Table S1). The SIDER1β sequences were divergent enough to be detected in the screening using the consensus sequence described by Bringaud F *et al*. [37] as a query. This divergence was confirmed by phylogenetic analysis, particularly with respect to *Tb*SIDER1 elements (Additional file 1: Figure S2).

**Figure 3:**
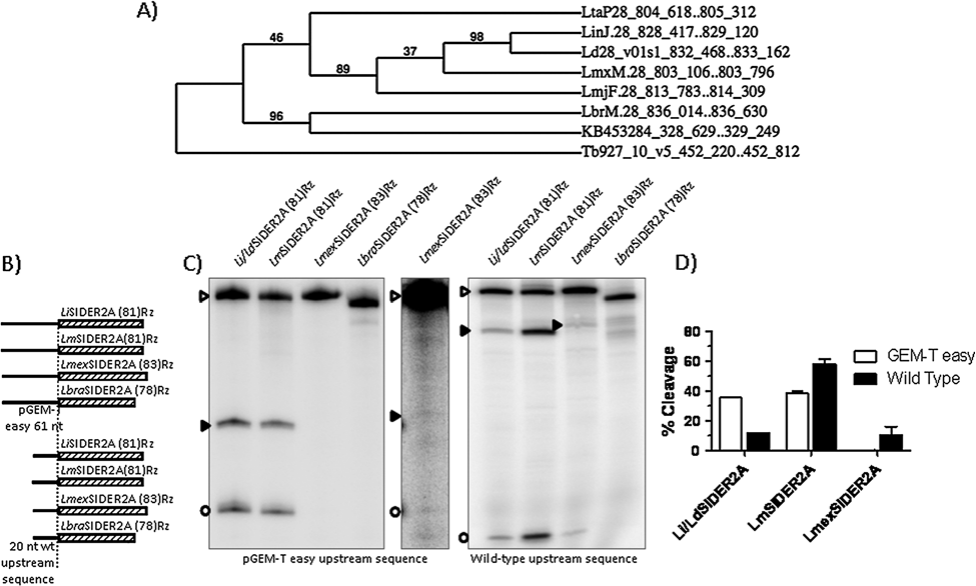
**Co-transcriptional cleavage of** ***Leishmania spp*** **ribozymes.** The cladogram for the *Leishmania* SIDER2 syntenic insertion is shown in **(A)**. The branch support values are given in %. The RNA constructs assayed are shown in **(B)**. The dotted line indicates the cleavage point and the striped boxes indicate the ribozyme region. The result of co-transcriptional cleavage reactions is shown in **(C)**. Note that the central panel is an overexposed version of the left panel *Lmex*SIDER2A(83)Rz (line 3). The quantification of independent triplicates is shown in **(D)**. Empty arrowheads indicate uncleaved products; solid arrowheads indicate the cleavage 3′ fragments. The empty circles indicate the cleavage 5′ fragments.

The position of each mobile element selected for the study was localised on a genomic map (Additional file 1: Figure S3). The existing synteny in *Leishmania spp* SIDER2 insertions indicates that these insertions were selected in the common ancestor of all the species. Actually, the cladograms obtained from both insertions revealed a similar divergence to that previously described [39, 40] for *L. infantum, L. donovani, L. major, L. mexicana* and *L. braziliensis* (only present in insertion 1) (Figure 3A). The low robustness offered by the branching support value between *L. mexicana* and *L. major* in the insertion 1 cladogram (<50, Figure 3A), compared to that obtained for a different branching in the insertion 2 cladogram (97, Additional file 1: Figure S2C), points to the latter being more reliable (according to [39, 40]). No consensus was reached for the position of *L. tarentolae*, which appeared in different positions in the cladograms for insertion 1 and 2, and always associated with a weak robustness branching support value (Figure 3A and Additional file 1: Figure S2C). Moreover, its position relative to *L. braziliensis* could not be determined due to a gap in the genome sequencing that partially affects *L. tarentolae* insertion 1 and completely affects *L. braziliensis* insertion 2 and its neighbouring region.

The localisation of *Leishmania spp* SIDERs in the 3′-UTRs has been previously reported, suggesting that these sequences may play a regulatory role [33]. Moreover, it has recently been shown that some of these sequence repeats promote the downregulation of the mRNA where they reside via endonucleolytic cleavage prior to deadenylation [35]. The existence of a ribozyme in *Leishmania spp* SIDERs may be involved in this cleavage.

### Detection of co-transcriptional in vitro activity of the selected candidates

**Figure 4:**
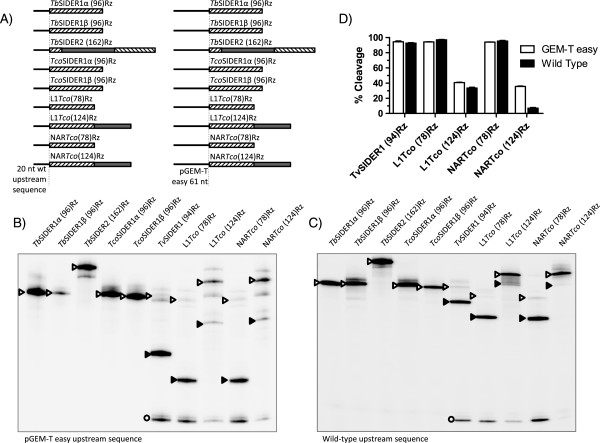
**Co-transcriptional cleavage of** ***Trypanosoma spp*** **ribozymes.** The assayed RNA constructs are shown in **(A)**. The striped region indicates the ribozyme region/s, and the grey regions indicate those that do not, *a priori*, contribute to ribozyme function (such as the large hairpin in *Tb*SIDER2(162)Rz or the attenuator sequences in L1/NAR*Tco*(124)Rz). The results of co-transcriptional cleavage reactions with the unrelated pGEM-T easy 61 nucleotides and the natural upstream 20 nt-long sequence are shown in **(B)** and **(C)** respectively. The different cleavage products are presented using the same code as in Figure 3. The co-transcriptional cleavage quantification of the active ribozymes, performed in triplicate, is shown in **(D)**.

Co-transcriptional cleavage activity was measured by resolving the transcription reactions of each ribozyme in denaturing polyacrylamide gels as previously described for L1TcRz [25]. Since the cleavage point is expected to be located on the 5′-side of the +1 nucleotide of each ribozyme [23, 25, 41], the addition of an upstream sequence to the transcription template allowed a cleavage to be detected via its two products: that of the upstream sequence region and the ribozyme catalytic sequence (Figures 3B and 4A, the dotted line indicates the cleavage point). To analyse the influence of the genomic sequence naturally located upstream of each ribozyme on ribozyme cleavage efficacy, two DNA templates were generated for each ribozyme by PCR using specific primers: one bearing the sequence corresponding to the 20 nt-long region naturally located upstream of each ribozyme, and one bearing the 61 nt-long unrelated region from the pGEM-T easy vector (see Figures 3B and 4A, in which they are referred to as the wild type upstream sequence and the pGEM-T-easy upstream sequence respectively). These were fused to the Pr77 sequence of each organism and employed *in vitro* transcription assays as indicated in Methods. Figure 2 shows the putative ribozymes selected for the *in vitro* transcription study, together with their acronym according to the trypanosomatid species and type of element to which they belong, and their nucleotide length. Transcription reactions were performed at 37°C for 2 h. Co-transcriptional cleavage activity was detected in *L. infantum*, *L. donovani*, *L. major*, *L. mexicana*, *T. vivax* and *T. congolense* for ribozymes *Lm*SIDER2A(81)Rz, *Li/Ld*SIDER2A(81)Rz, *Lmex*SIDER2A(83)Rz, *Tv*SIDER1(94)Rz, L1*Tco*(78)Rz and NAR*Tco*(78)Rz (Figures 3 and 4). No *L. tarentolae* ribozyme was assayed due to a partial sequence gap affecting signature I of insertion 1 of SIDER2 in the released genome, and a severe structural disruption in the signature I folding of insertion 2 of SIDER2 (Additional file 1: Figure S1). Signature I of *L. panamensis* SIDER2 at insertions 1 and 2 had severe structural disruptions (Additional file 1: Figure S1). Consequently these hallmarks were not assayed for HDV ribozyme activity either. The data show the ribozyme present in SIDER2ARz from *L. infantum*, *L. donovani*, *L. major* and *L. mexicana* to be functional, but not that from *L. braziliensis* (Figure 3C).

The upstream and downstream regions flanking HDV-like ribozymes have been shown capable of influencing ribozyme catalytic activity [42]. The present data indicate that the sequence upstream of the ribozyme region may do this (Figures 3 and 4). The cleavage activity of *Li/Ld*SIDER2A(81)Rz and *Lm*SIDER2A(81)Rz was moderate when the assayed upstream sequence was the 61 nt-long sequence upstream of the pGEM-T easy vector cloning site (see Figure 3C for activity and 3D for quantification of the cleavage products of the constructs shown in Figure 3A). In contrast, *Li/Ld*SIDER2A(81)Rz activity was reduced and *Lm*SIDER2A(81)Rz increased when the 20 nt-long wild type upstream sequence was assayed (see Figure 3C for activity and 3D for quantification of the cleavage products of the constructs shown in Figure 3A). *Lmex*SIDER2A(83)Rz activity was hardly detectable when combined with its wild type upstream sequence, and undetectable when the assayed upstream sequence was the pGEM-T easy vector (Figure 3C and D). Despite the high level of conservation between the *Lm*, *Li* and *Lmex*SIDER2A ribozymes and their upstream sequences, no clear pattern of misfolding interactions could be established that might justify the different influence exerted by the natural 20 nt-long and the unrelated 61 nt-long upstream sequences. The cleavage activity of native *Lmex*SIDER2A(83)Rz was very low compared to the rest of the functional ribozymes tested. This is consistent with severe folding abnormalities due to a base pair mismatch for the C1A39 nucleotides and a short (3 bp–long) P2 helix (Figure 2).

The catalytic activity of the putative ribozymes in *Trypanosoma spp* revealed *Tv*SIDER2A(94)Rz, L1*Tco*(78)Rz and NAR*Tco*(78)Rz to be highly active, independent of the upstream sequence (Figure 4B and C; for quantification of the cleavage products see Figure 4D). It is worth noting that none of the assayed SIDER ribozymes from *T. congolense* and *T. brucei* showed catalytic activity. This was expected for *Tco*SIDER1α(96)Rz and *Tb*SIDER1α(96)Rz, which have severe folding abnormalities, but not for the *Tco*SIDER1β(96)Rz and *Tb*SIDER1β(96)Rz which *a priori* show adequate folding (Figure 2 shows their folding; Figure 4 shows the results of the functional assays). However, in this and other work (Carreira P, López MC. *et al.*, manuscript in preparation) on promoter/ribozyme functionality, active ribozymes were detected in L1*Tc*/*ingi* clade LINE members from *T. brucei* and *T. congolense*.

The effect on the activity of the L1*Tco* and NAR*Tco* ribozymes exerted by the region downstream of the Pr77 signature (L1*Tco*(124)Rz and NAR*Tco*(124)Rz constructs, see Figure 4A) was also examined since this region attenuates the catalytic activity of L1*Tc*Rz [25]. Although this inhibition may be the consequence of the rapid *in vitro* transcription velocity of the T7 RNA polymerase [42], it reveals the induction of an RNA conformational switch (Figure 1C). The results indicate that the 46 nt-long sequence naturally located downstream of the L1*Tco* and NAR*Tco* ribozymes inhibits their activity (constructs L1*Tco*(124)Rz and NAR*Tco*(124)Rz, see Figure 4B and C; compare lines 8 and 10 to 7 and 9 in Figure 4B and C; see Figure 4D for quantification of the cleavage products). This result is different to that obtained for the *T. cruzi* homologous L1*Tc* and NAR*Tc*, in which only the downstream region of L1*Tc* had an attenuating effect [25]. The inhibition detected with respect to L1*Tco* and NAR*Tco* is expected given the high degree of sequence conservation of the downstream region in both elements and their similarity to L1*Tc*.

The NAR*Tco* and L1*Tco* elements showed strong sequence homology. However, the sequences located upstream of the Pr77-hallmark in both elements are different, and only that of the selected NAR*Tco* insertion exerted an inhibitory effect. This inhibition occurred only in the NAR*Tco*(124)Rz construct (Figure 4C, compare line 10 to line 8; Figure 4D shows the quantification of the cleavage products); it was not seen for NAR*Tco*(78)Rz (Figure 4C, compare line 9 to line 7; Figure 4D shows the quantification of the cleavage products).

Even when they met folding requirements, the active ribozymes of *Leishmania spp* showed only moderate activity compared to those of *Trypanosoma spp* (40-60% cleavage compared to ~95%). *Leishmania* SIDERs seem to concentrate in the 3′-UTRs in some of the protein coding genes and to act as post-transcriptional regulation signals [33]. In this context, a very active ribozyme might promote mRNA decay without any regulatory control, while moderately active ribozymes might be regulated by *trans*-acting RNAs or protein factors etc., as has been suggested for some SIDER signatures [30, 35]. However, under the present experimental conditions, no *in vitro* ribozyme function was detected for any of the Pr77 hallmarks in the 3′-UTR region of the genes showing downregulated mRNAs [36] (data not shown) [35].

### Kinetic parameters of co-transcriptionally active HDV-like ribozymes

**Figure 5:**
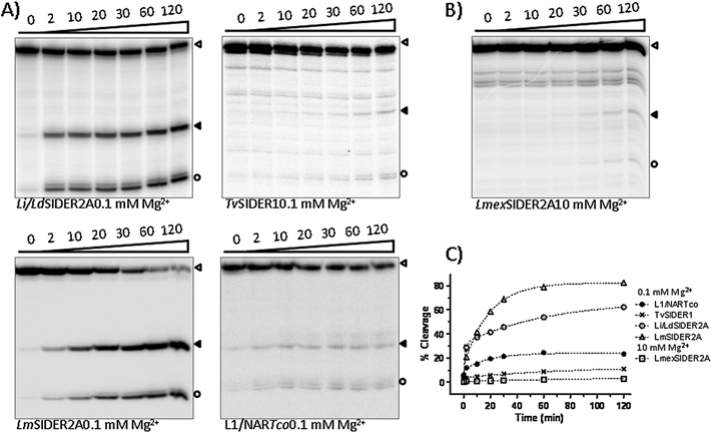
**Cleavage kinetics of trypanosomatid HDV-like ribozymes.** The uncleaved products of the transcription showed in Figure 3C (central panel) and 4D were gel-purified and subjected to cleavage reaction. Time course reactions at different Mg^2+^ concentrations were performed. *Li/Ld*SIDER2A(81)Rz, *Lm*SIDER2A(81)Rz, *Tv*SIDER1(94)Rz and L1/NAR*Tco*(78)Rz kinetic reactions at 0.1 mM Mg^2+^ are shown in **(A)**. The *Lmex*SIDER2A(83)Rz kinetic reaction at 10 mM Mg^2+^ is shown in **(B)**. The time course (min) of the reaction is shown above each line; the empty arrowheads indicate uncleaved products; solid arrowheads indicate cleavage 3′ fragments; the empty circle indicates the cleavage 5′-fragment. All the ribozyme reactions fitted the two-phase decay kinetic curve shown in **(C)**; the plotted data are the results of independent assays performed in triplicate. A more extended study is shown in Additional file 1: Figure S3.

Kinetic assays of the cleavage reaction were performed for each co-transcriptionally functional ribozyme carrying the 61 nt-long pGEM-T easy upstream fragment. Uncleaved products were gel purified and renatured in Mg^2+^-free buffer. The cleavage reaction started after the addition of MgCl_2_. Three different MgCl_2_ concentrations were assayed (0.1, 1 and 10 mM). The kinetic curves matched the two-phase decay model characteristic of HDV-like ribozymes (Figure 5 and Additional file 1: Figure S4).

Consistent with previous reports [42], the cleavage activity of these HDV-like ribozymes in *Trypanosoma spp* was greater under co-transcriptional conditions than after renaturation. The L1/NAR*Tco* and *Tv*SIDER1 ribozymes hardly cleaved after post-transcriptional renaturation, while their co-transcriptional cleavage rate was close to 100%. In contrast, *Leishmania spp* ribozymes showed greater cleavage efficiency at 2 h post-transcription than after 2 h of transcription (Table 1 and Additional file 1: Figure S5). This is consistent with a possible post-transcriptional regulatory function controlled by external factors. Nucleic acids chaperones or other RNA binding proteins might induce a refolding similar to that caused by heat shock prior to post-transcriptional cleavage (see Methods).

**Table 1:** **Kinetic parameters of cleavage reactions**

Ribozyme	R^2^	Two-phase		Plateau (%)				Co-transcriptional cleavage (%)
		Hyperbolic						
[MgCl_2_] mM	10	1	0.1	10	1	0.1	0	6
*Li/Ld*SIDER2A	0.9956	0.9932	0.9854	75.41	74.46	67.32	4.941	35.493 ± 0.402
	0.9379	0.9099	0.8769					
*Lm*SIDER2A	0.9184	0.9792	0.9975	73.58	61.54	82.37	-	38.622 ± 1.290
	0.8774	0.8956	0.9789					
*Lmex*SIDER2A	0.4263	-	-	3.764	-	-	-	0.179 ± 0.008
	0.3695	-	-					
*Tv*SIDER1	0.9690	0.8169*	0.8335*	99.27^!^	20.69	~14.62	~2.767	94.242 ± 1.076
	0.8924	0.7130	0.3603					
L1/NAR*Tco*	0.9817	0.9576	0.9789	69.74^!^	19.34	24.02	7.703	94.053 ± 0.181
	0.8749	0.6885	0.7805					

Note: *means an ambiguous fitting, and ! means data probably over-estimated. In all cases, the ribozyme kinetics fitted the R^2^ coefficient two-phase exponential decay model better than the hyperbolic model.

It is remarkable that for *Tv*SIDER1(94)Rz and L1/NAR*Tco*(78)Rz, the uncleaved product became progressively less common in the presence of 10 mM MgCl_2_ but with no accompanying accumulation of any cleavage products (Additional file 1: Figure S4). This may be an indication of some intrinsic instability of the uncleaved RNA at this Mg^2+^ concentration. Thus, the cleavage rate of both ribozymes at this concentration is possibly overestimated. Interestingly, *Lm*SIDER2A(81)Rz cleavage was more efficient at 0.1 mM than at 1 or 10 mM of MgCl_2_ (Additional file 1: Figure S4). It may be that an equilibrium displacement towards catalytic folding at 0.1 mM allows nearly all RNA molecules to achieve this state. At higher Mg^2+^ concentrations, non-catalytically active folding may be stabilised, preventing catalysis.

### Cleavage point determination in functional HDV-like ribozymes

To confirm the putative ribozyme folding, the accuracy of the cleavage point prediction was checked. The cleavage point was expected to lie on the 5′-side of the +1 nucleotide of the ribozyme domain shown in Figure 2. The downstream cleavage products (Figures 3 and 4, black arrowheads) obtained by the transcription of constructs using the 61 nt-long pGEM-T easy upstream sequence were then gel purified and used as templates for primer extension using radiolabelled specific primers that anneal at the 3′-end of each ribozyme. For *Lmex*SIDER2A(83)Rz, the template used for transcription was the natural 20 nt region located upstream of the ribozyme - the only one that allows catalytic activity.

**Figure 6:**
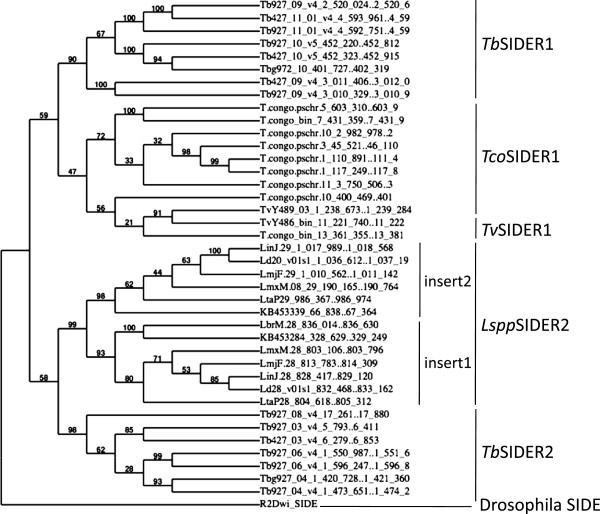
**Cladogram of trypanosomatid SIDER elements.** Cladogram produced by PhyML 3.0 aLRT software and TreeDyn 198.3 software. Branch support values are shown in %.

**Figure 7:**
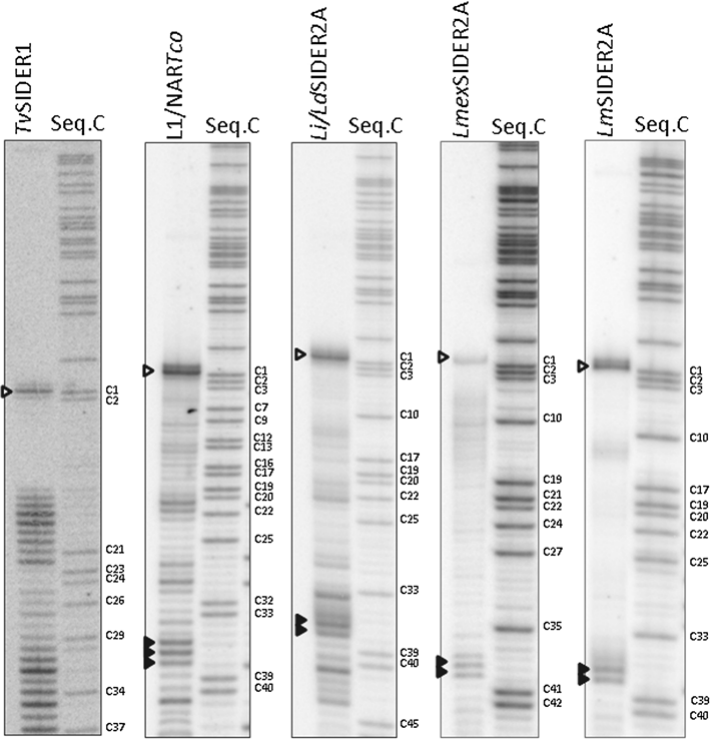
**Cleavage point determination by primer extension.** The figure shows the electrophoresis gels for the primer extension reaction using the cleavage 3′ fragment RNA of each ribozyme as a template, and a ribozyme-specific reverse primer. A cytosine-sequencing reaction for each ribozyme is shown to provide a size marker (entire gels are shown in Additional file 1: Figure S4). The empty arrowheads indicate the maximum extension product corresponding to the cleavage point. The solid arrowheads indicate the helix P1 internal extension stop.

To provide size markers, a sequencing reaction was performed using the primers employed in the primer extension assays. The maximum extension products from each primer coincided with the +1 nucleotide (C1) of the *Tv*SIDER1, L1/NAR*Tco*, *Li*/*Ld*SIDER2A, *Lmex*SIDER2A and *Lm*SIDER2A ribozymes (Figure 6 and Additional file 1: Figure S5, white arrowhead). Additionally, RT extension stops consistent with those previously described on the 3′ side of the helix P1 3′ strand [25] were detected in all ribozymes except for *Tv*SIDER1(94)Rz (Figure 7 and Additional file 1: Figure S4, black arrowheads). These stops were associated with a very tight junction composed of three consecutive GC pairs that would hinder polymerase progression (*Tv*SIDER1(94)Rz had only two GC pairs). Thus, it is possible that a C1A39 pair in *Lmex*SIDER(83)Rz contributes to the formation of a tight structure.

### Phylogenetic analysis of the origin of the L1Tc/ingi ribozyme

The existence of HDV-like ribozymes in related mobile elements within trypanosomatids, their relatively large size and their complexity, together suggest that these ribozyme sequences were vertically transferred. The same has been concluded by other authors [26] for the R2 retrotransposon ribozymes in insects. To examine this hypothesis, phylogenetic analysis was performed using the different isolated SIDER sequences. Alignment of the sequences involved in the HDV ribozyme structure within the Pr77 sequences of *Tb*SIDER2 and *LsspSIDER2* (which contain two Pr77 hallmarks in-tandem) and other SIDERs with Pr77 signatures of different length, required manual adjustment. These domains contain double stranded motifs and show sequence conservation that may have been promoted by selective pressure. The R2Dwi_SIDE sequence from *Drosophila willistoni*, which belongs to the R2 retrotransposon clade, was used as a known external sequence for the phylogenetic study of the analysed in-group (Figure 6). This sequence was chosen since R2Dwi_SIDE is also a short length SIDE element, and because it bears an HDV-like ribozyme at its 5′-end [3]. These features suggest R2Dwi_SIDE to be the closest relative to an HDV-like ribozyme-carrying element, yet it maintains enough genetic distance to be considered an outgroup.

The cladogram shows independent clades for the SIDER1 and SIDER2 elements. Within the SIDER1 clade, the *Tb*SIDER1 copies are clearly grouped. The *Tv*SIDER1 and *Tco*SIDER1 are also included within the SIDER1 clade and are closely related (Figure 6). *Lspp*SIDER2 and *Tb*SIDER2 are independently grouped within the same clade. The presence of a duplicated signature seems to have defined the differentiation of the SIDER1 and SIDER2 families before the radiation of the trypanosomatids. In this cladogram, the R2Dwi_SIDE clearly emerged as a group different to that in which the trypanosomatid SIDERs collected. However, this clearly points towards the existence of a common ribozyme ancestor. The ribozymes of the R2 LINE and HDV show strong nucleotide differences when compared to those found in the trypanosomatid organisms. These differences affect critical ribozyme structures such as the orientation of the two or three GC pairs in helix P1 and the number of base pairs and their constituent bases in the P1.1 pseudoknot (Additional file 1: Figure S7). The present results confirm that the sequence/structure requirements used by Webb CH. *et al*. [28] for HDV-like ribozyme screening were too restrictive; the present approach seems to be more flexible (Additional file 1: Figure S7). The results of the preliminary phylogenetic study performed here coincide with those of previous studies involving other molecular markers [39, 40] and support the idea that mobile elements as well as the extensive survey of retropositional events that might have occurred during the divergence among some eukaryotes may be used as a powerful method for making phylogenetic inferences [43, 44].

The HDV ribozyme belongs to a group of small catalytic RNAs with members that catalyse a similar self-cleaving reaction [45]. For 18 years the only two described HDV ribozymes were those observed in HDV RNA [46]. The high complexity of the HDV-like ribozymes explains why, until now, so few members have been described. The number of known HDV-like ribozymes is, however, now increasing, with examples even found in eukaryotic genomes ([23–28], Carreira P, López MC. *et al.* manuscript in preparation). The present work localises these ribozymes in a well-characterised and conserved sequence known as the Pr77-hallmark. The mobile nature of such retrotransposons may have promoted the spread of HDV-like ribozymes throughout trypanosomatids genomes.

## Conclusions

The Pr77-hallmark is a well-conserved sequence of the L1*Tc*/*ingi* clade retrotransposons in trypanosomatids. The present work describes HDV-like ribozymes in the Pr77-hallmark of mobile elements of *T. congolense*, *T. vivax*, *L. major*, *L. infantum, L. donovani* and *L. mexicana*. The existence of HDV-like ribozymes in these organisms may be a consequence of vertical transfer of a mobile element from a common trypanosomatid ancestor. The ribozyme activity present in the largely immobile SIDER elements of *Leishmania spp*[17], which are pervasively located in 3′-UTRs [33, 35, 38], suggests the occurrence of an exaptation event that turned these mobile elements into regulatory sequences.

## Methods

### Screening of HDV-like ribozyme candidates related to trypanosomatid mobile elements

The genomes of trypanosomatids held in the Eukaryotic Pathogen Resources Database (EuPathDB v2.15 31Aug12, http://www.eupathdb.org) were screened for the Pr77-hallmark of the L1*Tc*/*ingi* clade. These were probed with the Pr77 sequence from L1*Tc* and *ingi,* and with those from the *Tb*SIDER1 consensus sequences identified in African trypanosomes [37]. In order to select only homologues of Pr77 related to mobile elements, each hit was screened for a poly-A track 0.6 or 5–6 kb downstream of the 77 nt signature (see diagram of short and long elements in Figure 1), and for a recognisable TSD of 7–11 nt partially or totally conserved at both ends. Each Pr77-hallmark in a mobile element repeat was manually folded into an HDV-like ribozyme structure using predicted L1*Tc*Rz and *ingi*Rz foldings as guides. Screening was stopped when a candidate showing HDV ribozyme-like folding was found. The selected hits were used as queries to detect other homologues that might have escaped the original screening due to some major divergence from the Pr77-hallmark.

Large repeats (of around 5 kb) were annotated as DIRE elements (L1*Tco*, L1 from *T. congolense*). Short elements showing high homology to the L1*Tco* 5′ and 3′ ends were annotated as NAR*Tco*. Finally, short elements in *Leishmania* and *Trypanosoma spp* showing a single sequence with homology to the Pr77-hallmark were annotated as SIDER1 and those bearing two in tandem Pr77-hallmark as SIDER2.

### Synthesis of transcription templates for the different ribozymes

Unmodified and desalted primers were synthesised by Isogen Life Sciences at the 0.01 or 0.05 μmol scale depending on the primer length. The primers were designed to partially anneal to, but to cover the full length, of each ribozyme. A PCR reaction was performed with 100 pmol equimolar mix of each primer pair using 2 U Taq DNA polymerase (*Biotools B&M Labs*), 250 μM of each dNTP and 1X reaction buffer in a final volume of 50 μl. The PCR conditions were 95°C for 2 min (x1); 95°C for 30 s, Tm for 30 s, 72°C for 30 s (x5); 95°C for 30 s, 65°C for 30 s, 72°C for 30 s (x25); and 72°C for 7 min (x1). The melting temperatures (Tm) for the initial cycles were calculated based on the overlapping sequence of each primer pair by OligoCalc [47]. PCR products were gel-purified and ligated into the pGEM-T easy vector in the sense direction downstream from the T7 polymerase promoter.

The sequence of the primer pairs for each construct is shown in Additional file 1. For the *Tb*SIDER2(162)Rz construct, the primers were 5′TbSIDER2f and 3′TbSIDER2r; for *Tb*SIDER1α(96)Rz the primers were 5′TbSIDER1f and 3′TbSIDER1r; for *Tb*SIDER1β(96)Rz they were 5′TbSIDER1betaF and 3′TbSIDER1betaR; for *Tv*SIDER1(94)Rz they were 5′TvSIDER1f and 3′TvSIDER1r; for *Tco*SIDER1α(96)Rz they were 5′TcoSIDER1f and 3′TcoSIDER1r; for *Tco*SIDER1β(96)Rz they were 5′TcoSIDER1betaF and 3′TcoSIDER1betaR; for L1*Tco*(78)Rz and NAR*Tco*(78)Rz they were 5′L1Tco/NARTco-f and 3′L1Tco/NARTco-r; for *Li*SIDER2A(81)Rz they were 5′LiSIDER2Af and 3′LiSIDER2Ar; and for *Lbra*SIDER2A(83)Rz they were LbraSIDER2Af and LbraSIDER2Ar.

The *Lm*SIDER2A(81)Rz and *Lmex*SIDER2A(83)Rz constructs were generated by PCR using Taq DNA polymerase (*Biotools B&M Labs*) and employing 50 ng of the pGEM-T easy *Li*SIDER2A(81)Rz construct as a template plus primer pairs LmSIDER2Af and LmSIDER2Ar, and LmexSIDER2Af and LiSIDER2Ar, respectively.

To include the downstream sequences for L1*Tco*(124)Rz, new PCR amplifications were performed using 50 ng DNA of the pGEM-T easy L1*Tco*(78)Rz construct as a template and primer pairs 5′L1Tco/NARTco-f and 3′L1Tcord. The PCR product was gel-purified and ligated into the pGEM-T easy vector. The resulting construct was subsequently used as a template in a new round of PCR to generate NAR*Tco*(124)Rz with the primers 5′L1Tco/NARTco-f and 3′NARTcord. A pGEM-T easy construct was also generated for this product.

Templates for transcription carrying each ribozyme preceded by the unrelated 61 nt-long pGEM-T easy plasmid sequence were generated by PCR using the M13-20 forward universal primer and the specific antisense primer used for the generation of each construct. The PCR conditions were: 95°C for 2 min (x1); 95°C for 30 s, 52°C for 30 s, 72°C for 30 s (x25); and 72°C for 7 min.

Templates for transcription carrying each ribozyme, preceded by the natural 20 nt upstream sequence, were generated by PCR using each pGEM-T easy construct as a template and the primer pairs 5′T7-20TbSIDER2f and TbSIDER2r; 5′T7-20TbSIDER1f and TbSIDER1r; 5′T7-20TbSIDER1betaF and TbSIDER1betaR; 5′T7-20TvSIDER1f and TvSIDER1r; 5′T7-20TcoSIDER1f and TcoSIDER1r; 5′T7-20TcoSIDER1betaF and TcoSIDER1betaR; 5′T7-20L1Tcof and L1Tco/NARTco-r; 5′T7-20NARTcof and L1Tco/NARTco-r; 5′T7-20L1Tcof and 3′L1Tcord; 5′T7-20NARTcof and 3′NARTcord; 5′T7-20LiSIDER2f and LiSIDER2Ar; 5′T7-20LmSIDER2Af and LmSIDER2Ar; 5′T7-20LmexSIDER2Af and LiSIDER2Ar; and 5′T7-20LbraSIDER2Af and LbraSIDER2Ar. The sequence of all the listed primers is available in Additional file 1. The PCR conditions were: 95°C for 2 min (x1); 95°C for 30 s, 42°C 30 s, 72°C for 30 s (x5); 95°C for 30 s, 52°C for 30 s, 72°C for 30 s (x25); and 72°C for 7 min. All the transcription templates were agarose gel-purified by phenolic extraction and precipitation.

### Co-transcriptional cleavage assays

22 ng of PCR templates were transcribed using the T7 RNA polymerase kit (*PROMEGA*) in a final reaction volume of 10 μl at 37°C for 2 h, as previously described [25]. 10 μl of 2X loading buffer [94% (v/v) deionised formamide, 0.025% (w/v) xylene cyanol, 0.025% (w/v) bromophenol blue and 17 mM EDTA] were added to each reaction as a stop buffer. Samples were resolved by 8% polyacrylamide, 7 M urea, TBE 1X gel electrophoresis. Gels were dried and incubated with phosphor-storage screens for scanning in Typhoon 9400 (*Amersham Biosciences*) and quantifying using ImageQuant software (*Amersham Biosciences*).

### Cleavage reactions

Uncleaved transcription products from the 61 nt-long pGEM-T easy upstream sequence templates were separated in and eluted from polyacrylamide gels. These transcription reactions were performed with 45 ng of PCR DNA templates using the T7 RNA polymerase kit (*PROMEGA*) in a final reaction volume of 100 μl, following the manufacturer’s instructions with slight modifications (the UTP final concentration was reduced to 0.4 mM and 40 μCi of αP^32^UTP were added to radiolabel the molecules). After 2 h at 37°C, the reaction was stopped by adding 100 μl 2X loading buffer, and the uncleaved products were separated in 8% poly-acrylamide, 7 M urea, TBE 1X gels. The desired product was localised by autoradiography and eluted from the gel by shaking overnight in buffer TEN_250_ (10 mM pH 7 Tris–HCl, 1 mM EDTA, 250 mM NaCl) at 4°C followed by phenol extraction and precipitation.

Trace amounts of uncleaved radiolabelled RNAs were renatured in 0.5 pH7 Tris–HCl, 0.05 mM EDTA by incubation at 85°C for 5 min followed by 25°C for 10 min. Samples were then incubated at 37°C for 2 min. The reaction was started by adjusting the reaction buffer to 40 mM pH 7 Tris–HCl, 10 mM NaCl and final concentrations of either 10, 1, 0.1 and 0 mM MgCl_2_. Note that final concentration of EDTA was 0.02 mM.

10 μl aliquots were taken at different times. The reaction in each time point aliquot was stopped by adding one volume of 2X loading buffer and then maintained at –80°C. An aliquot was taken just prior to the addition of the reaction buffer (time 0). Reactions were performed in triplicate and resolved by 8% polyacrylamide, 7 M urea, 1X TBE gel electrophoresis. The gels were processed as described for co-transcriptional cleavage assays.

Data fitting was performed using Prism 5 v.500 software (*GraphPad Software, Inc.*). The double exponential equation was: *f*_*c*_ *= A + B*e^*–k1t*^ *+ C*e^*–k2t*^, where *f*_*c*_ is the cleaved fraction, *t* is time, *A* the cleavage fraction at infinite times, −*B* and *–C* the amplitudes of the observable phases, and *k*_*1*_ and *k*_*2*_ the observed first-order rate constants for the fast and slow phases respectively.

### Cleavage point localisation

Using the T7 RNA polymerase kit (*PROMEGA*), cleavage 3′-fragments for each active ribozyme were generated using 45 ng of the PCR DNA templates in a final reaction volume of 100 μl, following the kit manufacturer’s instructions. RNA products were resolved by 8% polyacrylamide, 7 M urea, TBE 1X gel electrophoresis. The desired fragments were localised by UV-shadowing and eluted from the gels. Elution was performed by shaking overnight in buffer TEN_250_ (10 mM pH 7 Tris–HCl, 1 mM EDTA, 250 mM NaCl) at 4°C and followed by phenol extraction and precipitation. The PCR DNA templates used were those bearing the 61 nt-long pGEM-T easy upstream sequences, with the exception of *Lmex*SIDER2A(83)Rz. This cleavage reaction was only detectable when the natural sequence upstream 20 nt was included in the template. The yield of the cleavage reaction was too low to detect the RNA fragment by UV-shadowing, so excision was performed using products of known size as markers to suggest where the fragments may lie.

The cleavage point was mapped by primer extension using the isolated RNA as a template. Primers were radiolabelled by phosphorylation using T4 PNK (*Roche*); this reaction involved 15 pmol of primer, 10 units of PNK and 40 μCi of γ^32^P-ATP in a final volume of 10 μl. The reaction was incubated at 37°C for 30 min and the products purified in 20% polyacrylamide, 7 M urea, TBE 1X gels as described above for the uncleaved RNA in cleavage reactions. The labelled primers were 3′LiSIDER2Ar (for *Li*SIDER2A(81)Rz and *Lmex*SIDER2A(81)Rz), 3′LmSIDER2Ar, 3′L1Tco/NARTco-r and 3′TvSIDER1r

Reverse transcription was performed using AMV reverse transcriptase (*PROMEGA*). Primer and RNA annealing was performed with 200 μg of each RNA template, approx. 1.8 pmol radiolabelled primer, and 20 U RNase-in plus (*PROMEGA*) in a final volume of 11 μl at 70°C 5 min, employing a temperature reduction ramp of 2°C/20 s until reaching 42°C. Extension reactions were performed by adding 1 mM (final concentration) of each dNTP, 40 U RNase-in plus (*PROMEGA*), 12.5 U AMV reverse transcriptase, and reaction buffer 1X, in final volumes of up to 25 μl and incubating at 42°C for 60 min.

Manual sequencing of the corresponding pGEM-T easy constructs for each primer extension was performed using the same primer and employing the Thermo Sequenase™ Cycle Sequencing Kit (*USB*), following the manufacturer’s instructions. Primer extension and sequencing reactions were resolved by 8% polyacrylamide, 7 M urea, TBE 1X sequencing gel electrophoresis. Results were obtained as described above for the co-transcriptional cleavage assays.

### Phylogenetic analysis

For the phylogenetic analysis of the sequences of the elements *Tb*SIDER1, *Tb*SIDER2 and *Tco*SIDER1 (Additional file 1: Figure S2), the sequence *Lspp*SIDER2A was used as an outgroup; while *Lspp*SIDER2A elements were aligned using *Tb*SIDER1α as an outgroup (Figure 4A). Sequence alignment was performed using ClustalW2 software [48]. Phylogenetic analysis was performed using PhyML 3.0 aLRT software (from Phylogeny.fr); and the Likelihood-Ratio Test (aLRT) and with the default settings for DNA/RNA (SH-like test and the HKY85 substitution model). Likelihood-Ratio Test is preferred when only nucleotide variations have likely occurred through the evolution [49–51]. Cladograms were produced using TreeDyn 198.3 software (from Phylogeny.fr). For this, the SIDER sequences were employed without the TSDs (sequences in bold in Additional file 1: Figure S1).

For the phylogenetic analysis of the sequences of the SIDER elements of the whole clade (Figure 6), Pr77 signatures and the SIDER bodies of the elements were aligned separately, using Clustal W2 software. Pr77 sequences were manually curated to align ribozyme structural regions (Additional file 1: Figure S6), and then both Pr77 and the rest of the SIDERs were put together in the same alignment (see Availability Supporting Data below). Phylogenetic cladogram was obtained using PhyML 3.0 aLRT software (from Phylogeny.fr); and the Bootstrapping procedure employing the default settings for DNA/RNA (100 bootstraps and the HKY85 substitution model). A parsimony analysis like that is preferred when gain or loss of domains have occurred, for instance the Pr77-hallmark duplication [49–51]. Cladogram was produced also using TreeDyn 198.3 software (from Phylogeny.fr).

### Availability of supporting data

The cladogram in Figure 6 and its related matrix and alignment are available at the TreeBASE public repository http://purl.org/phylo/treebase/phylows/study/TB2:S15572.

## Electronic supplementary material

Additional file 1: **Further information regarding HDV-ribozymes in Trypanosomatids.** (DOCX 2 MB)

## Abbreviations

LTR: Long terminal repeat
TPRT: Target-primed reverse transcription
ORF: Open reading frame
LINE: Long interspersed nuclear element
SINE: Short interspersed nuclear element
DIRE: Degenerated *ingi*/L1*Tc*-related element
SIDE: Short internally deleted elements
SVA: SINE-R, VNTR and Alu
HDV: Hepatitis delta virus
NARTc: Non-autonomous retroposon in *Trypanosoma cruzi*
NARTco: Non-autonomous retroposon in *Trypanosoma congolense*
RIME: RIbosomal mobile element
SSR: Strand switch region
SIDER: Short interspersed degenerated retroposon
UTR: Untranslated region
AMV: Avian myeloblastosis virus
PNK: Polynucleotide kinase.
