# Supplementary material for: The wide expansion of hepatitis delta virus-like ribozymes throughout trypanosomatid genomes is linked to the spreading of L1Tc/ingi clade mobile elements
Source: BMC Genomics. 2014 May 6;15(1):340. doi: 10.1186/1471-2164-15-340 (PMC4035085; doi:10.1186/1471-2164-15-340)
Supplement: Supplementary file 1 — Additional file 1: Further information regarding HDV-ribozymes in Trypanosomatids. (DOCX 2 MB) [file 12864_2013_6063_MOESM1_ESM.docx]

**Additional file 1: Further information regarding HDV-ribozymes in Trypanosomatids.**

SEQUENCES ISOLATED FROM TRYPANOSOMATIDS

The sequence of each mobile element is depicted in bold. The Pr77 signature is underlined.

*Trypanosoma brucei* SIDER2 sequences:

Tb927_08_v4:17,261..17,880.

GTTTGTAAATATGCTCATTGGC**CCCTGGTTTAGTGGTAAAGTAGAGGTCTCGTCGCATATAGTACCGGCATCGATCCCCGTGGGCGGAAAGTATTTAGCTTACACTAAGGATGGATCTGCCTCAACGTGGCGCCAGGGCCCAGTACAAGAAGAGAAATCGGCTGGGAAGTCAAATGTCCCATCGAAACAGCGGCTGCAACACCGGATATGGTGCGACCGCTCCGTGGGGAGAACCATTACTTAGCTTTCCACGGGCCAATACTCTGCTCAGGTGGGAGAACCAAAATCCCTGATACAACCTCCGTCCGGGGAGGCAGATGGATATGGTAAACATGGTTCCAACGCAACGTGTGTGTGGGGAGTCGAACTGGCGTCAAATGGAGACCGCCCCCCTAGACAAGCGCACATGCGGTCGGCCTCCAGTTGGTATCAATTCTCAAAAAAAGGGGGTTTAGTGGGTAGGAACGCCGGTATCCAGTGTTGAGTCCCACGCTACACCATGTCATCGCGGCGTGGTGTGCTTAAAAGCATTTACTTCTTACCATTGGGTGGAGCGATTGCATGGTGCTGACATCTGCGCGAGGGAAAATAAAAAAAACA**CTGTGTTTCCTCCTGGCGGT

Tb427_03_v4:6,279..6,853

GCTGGCTATTATATTCGGCTTA**CGCTGGTTTAGTGGTTAAGTAGATTGGTCGTCGCAGATAGTACCGGAATCGATCCCCGCGGCCAGCGAGTATTTAGCTTACACTAAGGATGGATCTGCCTCAACGTGGCGCCAGGGTCCAGTACCAGAAGAGAAATCGACTGGGAAGCCAAATGTTCCATCCAAANGGCGGCCGCAACACCGGATATGGTGCGACCGCTCCGTGGGGAGAGAACCATTACTTAGTGTTCCACGGTCCAGTACCCTGCTCAGGTGGGGGAACCAAAATCCCTGATACAACTTCCGTCCGGGGAGGCAGATGGATATGGTAAACATGGTTCAGACGCAACGTGGGGGGAATCGAACTGGCGTCAAGTGGAGACCGCCCCCCTAGACACGCGCACATGCGGTCCCCCACCCGTGTGTATAAATTCTCTAAAAAACAAGGGAGTTTAGTGAGTAGGAATGACAATCTCCAGCGTTGAGTCTCACACTACCACGTTCTCGTGGTGTGGTGTGCGTAAATGCATTTNCTCTTTACCCTCGTGTAGACCGACAGCATGGTCTTGAAGGTTGCGCGAGGAAAAATCAAAAAAA**TTTNTACTGCCGCTTTTTCTTT

Tb927_03_v4:5,793..6,411 (TbSIDER2)

GTTGTCTTTCATCTAATCGGTG**CCCTGGTATAGTGGTTGAGTAGAGTGCTCGTTGCTGATAGTGCTGGCATCGATCCCCGTGGGCGGCGAGTATTTAGCTTACACTACGGATGAATCTGCCTCAACGTGGCGCCAGGGTCTAGGACCAGAAGAGAAATCGACTAGGAAGGCAAATGTTCTATCCAAAAGGCTGCCACACCACAGGATATGGTGTGACCGCTCCGTGAGGAGAGAACCATTACTTAGTGTTCCACGGTCCAGTACGCTGCTCAGGTGAGGGAACAGAAATCCCTGATACAACTTCCGTCCGGGGAGGCAGATGGATATGGTAAATATGGTTCAGACGCAACATGAGGGGAATCGAACTGGCGTCAAATGGAGACCGCCACCCTAGACACGCGCACATGCGGTCCCCCACCCGTGTGTATAAATTCTCTAAAAAACAAGGGAGTTTAGTGAGTAGGAATGACAATCTCCAGCGTTGAGTCTCACACTACCACGTTCTCGTGGTGTGGTGTGCGTAAATGCATTTACTCTTTACCCTCGTGTAGACCGACAGCATGGTCTTGAAGGTTGCGCGAGGAAAAATCAAAAAAA**TTTTTTACTGCCGCTTTTTCTT

Tb927_06_v4:1,550,987..1,551,601

AACGAACACACTCTTCTTCGCA**CCCTTGTTTGGTGGTTAAGTAGAGTGCTTGTCGAACATTGTACAAGGGTAGATCCCAGGGGGCGGAGAGTATCTAGCTTACACTAAGAATGAATCTGCCTCAATGTGGCGCCAGGGTCACGTACCAGAAGAGAAATCGACTGGAAAGCCAAATGTTCCATCCAAAAGTCGGTTGCAATACTGAATATGCTGTGGCTGCTCCATGAAGAAAGAACCATTACTTAGTGTTGCACCACGGTTCAGTACCCTGCTCAGGTGGGGGAACCAAAATCCCTGACCCAACTTCCGGCCGGGGAGGCAGATGGATATGGTAAACATGGTTCAGATGCAATGTGGGAGGGATCGAATGGGCGTCAAGTGGAGACCACCCCCCTAGACACGCACACATGCGGTCTCTCACCCGTGGGTATCAATTCTCCAAAAAACAGGGAATTTCAGTGGGTAGGCACGCTGGTCTCCAGCGCTGAGTCCCACACTACCATATCGTCGTGGCGTGGTGTGCGTAAATGCATTTCCTCCTTACCATCGGGTGGACAGACTGCATGGTCCTGAAGGTTGCGCGAGGGGAAAAA**GTAACGGTTGATTCTATCTTTA

Tb927_06_v4:1,596,247..1,596,869

CTACAACAGCAACTTAACCGGC**CCCTGGTTTAGTGGTTAAGTAGAGTGCTCGTCGCAGATAATTCCTGGGTGAATCCCCGCGGGCGCAGATTATTTAGCTTACACTAAGAATGAATCTGCCTCAATGTGGCGCCAGGGTCACGTACCAGAAGAGAAATCGACTGGAAAGCCAAATGTTCCATCCAAAAGTCGGTTGCAATACTGAATATGCTGTGGCTGCTCCATGAAGAAAGAACCATTACTTAGTGTTGCACCACGGTTCAGTACCCTGCTCAGGTGGGGGAACCAAAATCCCTGACCCAACTTCCGGCCGGGGAGGCAGATGGATATGGTAAACATGGTTCAGATGCAATGTGGGAGGGATCGGATGGACGGCAAATGGACAACGCCCCCTAGAATCGCACACATGCGGTGGCCCACCTGTATGTGTCGATTCTCAAAGAAACAAAGGGGGTTTTAGTGGATAGGCACGCTGGCCTCCAGCGTTGAGTCCCACACTACCATGTCCTCACGGCGTGGTGCGGATAAATGCATTTAAGCCTTACCCTCGGCTGGTCAGACTGCATGGACCTGACGGTTGCTCGGTGGAAAATTTAAAAAA**GTCAACGAGTATAGAATTTTAA

Tb927_04_v4:1,473,651..1,474,277

GTAGGTATGTTCTTCCGCTGTA**CCCTGGTCTAGTGGTCAAGTAGCGTCCGCGTCACAGATAGTACAGGGATCGGTCCCCGCGGCCGGCGAGTATTTAGCTTACACTAAGGATGAATCTGCCTCAACGTGGCGCTAGGGTTCAGGAACAGAAAAGAAATAAACTGGGAAGTCAAATGTTCCATCCAAACGGCGGCCGCAACACCGGATCTGACGCGCCCGCTCCGTGGGGAGAGAACTATTACTAAGTATTCCACGGTCCAGCGCCCTGCTCTGGTGTGGGAACCCTAATCCCTTATACAACTTCCGTCCGGGGAGGCAGATGGATATGGTAAACATGGTTCAAACGCAACGTGGGAGGATCGAACGGGCGCCAAATGGAGACCGACCCCCTAGACACACGCACATACGGTCACCCAGCCGTGGGTGTCAATTCTCCAAGAAAAAAACAGGTGGTTTTAGTGGGTAGAAATGACAATCTCCAGAGCTGACCCCCACACTACCACGTTATCGTGGCGTGGTGTGCGTAAATGCATTTTCTCTTTGCCCTCGGGTGGACCGACTGCATGGTCCTGACGGTTGCGCGAGAGAAAATCAAATGAAAAAAA**TTGCTTTGTTCTTTGCGGTGCT

Tbg927_04:1,420,728..1,421,360

GTAGGTATGTCCTTCCGCTGTA**CCCTGGTCTAGTGGTTAAGTAGCGTCCGCGTCACAGATAGTGCAGGGATAGATCCCAGCTGGCGGAGAGTATTTAGCTTACACTAAGAATGAATCTGCCTCAACATGGAGTCAGCGTCCAGGGCCAGAAGAGAAATCAACTGGGAGGCCAAATGCTCCATCCAAACGGCGGCCGCAACACCGGATATGGTGTGACCGCTCCGTGGGGAGAGAACCATTACTTAGTGTTCCACCACGGTTCAGCACCCTGCCCAGGTGAGGGAACAGAAATCCCTTATACAACTTCCGTCCGGGGAGGCAGATGGATATGGTAAACATGGTTCAAGCGCAACGTGGGGGGAATCGAACGGGCGCCAAATGGAGACCGCCCCCCTAGACACACGCACATACGGTCACCCAGCCGTGGGTATCAATTCTTCAAAAAAAGAAAACTGAGGGTTTTAGTGGGTAGAAACGCCAGTATCCAGCGTTGAGTCACACACTACAATTTCGTTGTGGCATGGTGTGCGGAAGTGCATTCCCTCCTTACACTCGGGCAGACCGACTGCATGGTCCTGACGGTTGCGCGAGAGAAAATCAAATGAAAAAAA**TTGGTTTGTTCTCTGCGGCGCC

*Leishmania spp.* SIDER2 sequences:

Insertion 1.

LmjF.28:813,783..814,309 (LmSIDER2A)

TTCTTTCCTTCGCTCTGTCT**CCCTGATAACGGGGGACACCTCATCGTGGTATCAGGGTCCAGTACCCACTCTCTCTGTGGGGAAGCCAAGCAGCCCCTACTCCTGCCACTGCACAACCACCTCTGGTGGTGACAGGGTCAGGCGCGCATGACGTAGGGAGGTCAGAGCGATGTATCACTGCCGATGTCCGCAGTGTCCGGTCCTGGAGGGCGTGGCTGTGGGGCGACCTGCGGGGCGGGGGTGGGTACGGTTCGAGGCAGAAGCCATGCACCGATGACCGGGTCTGGGCATGGCAGCACCTCGTGTGCCTACGGCTGCCTCGCGCCGCGCGACGGGGCCTGTGACCGGCCGGGCAGAGATGAGCTTAGCTCTTGTGTGGCAGAGAGATGGGGACGACAAAGAAGTTCAGTCTCCTGTTCTCGTGTCTCAGGCTCCATGTACCTTGAGTGGAGTGTGCGGTTCGTAGATAGCATGACGGCGTGCGCTTGTTTGAACAGAGGAGCAAAA**CTGTCTGTATGGGACGAGCA

LinJ.28:828,417..829,120 (LiSIDER2A)

TTCTTTCCTTCTCTCTGTCT**CCCTGATAACGGGGGACACCTCAGCGTGGTATCAGGGTCCAGTACCCACTCTCTCTGTGGGGAAGCCAAGCAGCCCCTATTCCTGCCACTGCACAACCACCTCTGGTGGTGACAGGGTCAGGCGCGCGTGACGTAGGGACGTCAGAGCGATGTATCACTGCCGATGTCCGCAGTGTCCGGTGCTGGACGGCGTGGCGCCGGAGCGACCCGCGGCCGCGCACACGTTTTCGCCATCCACAGGATGGGCGGAGTGTCGGCGTGACTCGAACGCGTCCCACCCCCGGCCCTCACTGCCCACTGGGGGCGGGGTGAGCCTGGCCCCCCCCCCCCCGAGAGGGATGCCCCGGGTGATGGCCAGCATAATGTGCGTGGCTGTGGGGCGACCTGTGGGGCGGGGTTGGGTAAGGTTCGAGGCAGAAGCCATGCACCGATGACCGGGTCTGGGCATTGCTGCACCTCGTGTGCCTACGGCTGTCTCGCGCCGCGCGACGGGGCCTGTGACCGGCCGGGCAGAGATGAGCTTAGCTCTTGTTGTGTGGCAGAGAGGAGACGACAAAAAAATTCAGTTTCCGGTTCTCGTGTCTCAGGCACCATGTATCTTGAGTGGAGTGTGCAGTTTGTAGATAGCATGACGGCGTGTGCTTTTTTGAACAGAGGAACAAAA**CTGTCTGTATGGGACGAGCA

LmxM.28:803,106..803,796 (LmexSIDER2A)

TTCTTTCCTTCCCTCTGTCT**CCCTGATAACATGGGTGACACCTCAGCGTGGTATCAGGATCCAGTATCCACTCTCTCTGTGGGGAAGCCAAGCAGCCCCTATTCCTCCCACTGCACAGCCACCTCTGGTGGCGACAGGGTCAGGCGCGCATGACGTAGGGAGGTCAGAGCGACGCATCGCTGCCGATGTCCGCGGTCCTGTCCTGGACGGCGTGGCGTCGGAGGGGCCTGCCACCGCGCACGCGCTTGCACGACCCACTGGATGGGCAGAGTGTCGGCATGACTCGAACGCGCCCCACCCGGCCCTCGCTGCCCACTGGCGGGGTAAGGCTGGGGCACCCCGAGAGGGATGCCCTGGGCGATGGCCGGGATAATGCGCGCGGCTGTGGGGCGACCTGCGGGGCGGGGGTGGGCAAGGCTCGAGGCAGAAGCCATGCATCGATGACCGGGTCCGGGCATTGCTGCGCCTCGTGTGCCCACGGCTGCCTCGCGCCGCGCGACGGGGCCTGTGACCGGGCGGGCGGAGATGCGTTGAGGTCATGTGTGTGGGGATAGAGACGACAAAAAAAAATCAGTCTCCGGTTCTCGTGTCTCAGGCTCCATGTACCTTGAGTGGAGTGTGTGGTTCGTAGATAGCATGATGGGGTGTGCTTGCTTCAACGGAGGAACAAAG**CGGTCTGTATGGGGCGAGC

LtaP28:804,618..805,312

TTCTGTCCTTCACTCCGTCT**CTCTGATAACGGGGGACACCTCAGCGTGGTATCAGGGTCCANNNNNNNNNNNNNNNNNNNNNNNNNNNNNNNNNNNNNNNNNNNNNNNNNNNNNNNNNNNNNNNNNNNNNNNNNNNNNNNNNNNNNNNNNNNNNNNNNNNNNNNNNNNNNNNNNNNNNNNNNNNNNNNNNNNNNNNNNNNNNNNNNNNNNNNNNNNNNNNNNNNNNNNNNNNNNNNNNNNNNNNNNNNNNNNNNNNNNNNNNNNNNNNNNNNNNNNNNNNNNNNNNNNNNNNNNNNNNNNNNNNNNNNNNNNNNNNNNNNNNNNNNNNNNNNNNNNNNNNNNNNNNNNNNNNNNNNNNNNNNNNNNNNNNNNNNNNNAGCGTGCCTGTGGGCGACGTGCGGGGCGGGGTGGGTGCGGTTCGAGGAAGAGGCCATGCTCCGACGACTGAGTCTGGGCATTGCTGCGCCGCGTGTGCCTAATGCTGCCTCGCGCCACGCGACGGACGTGTAACCGGGGCCAGGCAGAGTGGGAGTTGAGCTCTTGTTGTGTGGCAGCATGGACACGTTGAAAGAAAATTCAGTGCCCGGCTCTTGTGTCTCAGGTTCCATGTTCCGAGAGTGGAATTTGTGGTTTGTAGATGTCGCGGCGTGTTGCTGGTTGAAAAGAGGAACAAAA**CTGTCTGTATAGTGTGAGCA

LbrM.28:836,014..836,630 (LbraSIDER2A)

GTTTCTGGCTTTCACTGTCG**CCCTGATGACGCGGAAAGGTCCTAGCGTGGTATCAGGGCCCGCCCCCCGCTCGGCGGGGAGGTCAGGCAGCCCCCTATCCCTGCCAATGCCGAACCGCCCTTGGCGATGGTAGGGACAGGTGTCTGAAAGACGAGGGGGGGGTCGTGGCGGCGTGTCGCTGCTGATGTCGGCGGTCCGGTTGTAGAGGGCGCTGCGTCGGTGCGGCCTGCGGCCGTGGGCGCGCCTGTGCCACCCGTGTGGTGAGCGGGTTGCTGGCGGCCAGCGTAATGGCCAGGGCTGCGAGGCTGCCCGGGGGACGGGGTCGAGTGGGTAGCGTTTGTGGCGGGGGTGGTGGTGGCGCCCGGGTGACTGAGTCGGCGCGTTGCTGTGGCGCGTGTCCCGCTGTTTCGTACCGGGCGATGGGCCTGCAGTGGGTTGGGTAAAGTGGAATGTAAGCTCGGGCTCTATGGCTGAATGGATGTTATAAAAAAAAGTGACTCTCTTCTACTCTCGTGTCCCAACCTGCATATACAATCAGGGGAATGAGTGGTTCGCATATCACGCGTCGGTGTGCGCTTGTTGGAACAGAGCAATAAA**GCTTTCTACATGGTCCGAGT

Ld28_v01s1:832,468..833,162

TTCTTTCCTTCTCTCTGTCT**CCCTGATAACGGGGGACACCTCAGCGTGGTATCAGGGTCCAGTACCCACTCTCTCTGTGGGGAAGCCAAGCAGCCCCTATTCCTGCCACTGCACAACCACCTCTGGTGGTGACAGGGTCAGGCGCGCGTGACGTAGGGACGTCAGAGCGATGTATCACTGCCGATGCCCGCAGTGTCCGGTGCTGGATGGCGTGGCGCCGGAGCGACCCGCGACCGCGCACACGTTTTCGCCATCCACAGGATGGGCGGAGTGTCGGCGTGACTCGAACGCGTCCCACCCCCGGCCCTCACTGCCCACTGGGGGCGGGGTGAGCCTGGGCCCCCCGAGAGGGATGCCCCGGGTGATGGCCAGCATAATGTGCGTGGCTGTGGGGCGACCTGTGGGGCGGGGGTGGGTAAGGTTCGAGGCAGAAGCCATGCACCGATGACCGGGTCTGGGCATTGCTGCACCTCGTGTGCCTACGGCTGCCTCGCGCCGCGCGACGGGGCCTGTGACCGGCCGGGCGGAGATGAGCGTAGCTCTTGTGTGGCAGAGAGGAGACGACAAAAAAATTCAGTTTCCGGTTCTCGTGTCTCAGGCACCATGTATCTTGAGTGGAGTGTGCAGTTTGTAGATAGCATGACGGCGTGTGCTTTTTTGAACAGAGGAACAAAA**CTGTCTGTATGGGACGAGCA

KB453284:328,629..329,249 (*Lpan*SIDER2A)

GTTTCTGGCTTTCACTGTCG**CCCTGATGACGAGGAAAGGTCCTAGCGTGGTATCAGGGCCCGCCCCCCGCTCGGCGGGGAGGCCAGGCAGCCCCCTATCCCTGCCAATGCCGAACCGCCCTTGGCGATGGTAGGGACAGGTGTCTGAAAGACGAGGGGGGGGGGAGTCGTGGCGGCGTGTCGCTGCTGACGTCGGCGGTCCGGTTGTGGACGGCGCTGCGTCGGTGCGCCCTGCGGCCGTGGGCGCGCCTGTGCCACCCGTGTGGTGAGCGGGTTGCTGGCGGCCAGCGTAATGGCCAGGGGAGCGAGGCTGCCCGGGGGACGGGGTCGGGTGGGTAGCGTTTGTGGCGGGGGTGGTGGTGGCGCCCGGGTGACTGAGTCGGCGCGTTGCTGTGGCGCGTGTCCCGCTGTTTCGTACCGGGCGATGGGCCTGCAGTGGGTTGGGTAAAGTGGAATGTAAGCTCAGGTTCTATGGCTGAATGGATGTTTTAAAAAGAAGTTACTCTCTTCTACTCTCGTGTCCCAACCTCCATATACAATCAGGGGAATGAGTGGTTCGCATATGACGCGTCGGTGTGCGCTTGTCGGAACAGAGCAATAAA**GCTTTCTACATGGTCCGAGT

Insertion 2.

LmjF.29:1,010,562..1,011,142

CCAACC**CCCTGATGGCGGGGGGATACCTCAGCGTGGTATCAGGGCCCAGTACCCACTCTGTGTGGAGAAGCCAAGCAGCCCCCCTATCCCGGTCAATGCATGACCACTTCCAGTGGTGGCAGAATCATGTACCTGCGACGTGTGGGGGGAGATTAGGGAGATGCATCGCTGCAAATATTGCCGGTGAAATCCTGGGCGACTTTGCGTTGGGGCCACCCGCGACAATGACCACGCTTGTACCATTCACGTGATAGGCGACGTGTCCGCGTGACTGGACCGTATCTTGCCCGAGCCTCACTGCCTGATGGCTGAGGCAGCCCGTGCGACCGCGCAGGAGATGCACCAGGTGGCGACCTGCATGATGGGGGCTGCTGCGCGGCGATCTTCGGTGCGGAGCGAGTAGTATTCGTTGCAGCGATGACTGGGTCTGCATTGTTGTAACGCGAGTGTCTACCGCTGCATTGCACCACACGATGGGGCCTGTGACAAGCGGTAGGGTGGGTTGAGTGGAGTTTCACTCATGTTGTACGGCAGAGAGAGAGAGATGGACACGTTGGGGGTAAGAGGTAAAAAA**GTGATCG

LinJ.29:1,017,989..1,018,568

CCAACC**CCCTGATGGCGGGGGGACACCTCAGCGTGGTATCAGGGTCCAGTACCCACTCTGTGTGGGGAAGCCAAGCAGCCCCCCTATCCCGGTCAATGCATAACCACTTCCGGTGGTGGCAGGACCATGTACCTGCGACGTGGGGGGGGAGATTAGGGCGATGCATCGCTGCTAATGTCGCCTTTGTGGTCCTGGGCGACTTTGCGTCGGAGCCACCCGCGACAGTGACCACGCTTGTACTACTCACATGATAGGCGACGTGCCCGCATGACGGGAGCGTATCTCACGCGGGCCTCACTGCCTGATGGCGGAGGTAGCCTGCGCGACCGCGCGGAGGATGCACCAGGTGACGATCTGCATGATGGGAGCTACTGTGCGGCGATCTTCGGTGCAGGGCGAATAGTATTCGTTGCAGGGATGACTGGGTCTGCATTCCTGTAACGCGAGTGTCTACCGCTGCATTGCACCACACGATGGGGCCTGCGGCAGGCCGTGGGGCGGGTTGAGTGGAATTTCACTCATGTTGTACGGCAGAGAGAGAGATGGACACGCTGGTGGTAAGAGGTAAAAAAAA**TGACTG

LtaP29:986,367..986,974

CCAACC**CCCTAATGACAGGGGGACACCTCGGTGTGGTATCAGAGTGCAGCACCTAACTCTCTATGGGGAAGCCAAGAATCCCCCCAATCCCTGTCAACGCACCACCACTTCAGCTAATGGTGGAACCATGTATGTGCGACATGGGGAGAGGAGATCAGAGAGATGCATCTCTACCGATATCGACAGTCATGTCATGCACTACGTTGCGCCGGAGCCACCTGCAACACTGAAGGTGCTTGCAGCATTTTCAGCAGATGTGAAGTGCCTGCGTCGCTCAAGCATATCTTCCCAGTCCTTGCTGCCTGATGGTGGAGGCAGCTTGTGCCACCGCGCGCGAGATACAGCAGGTGTCAGCCGGCGTGATGAGAGCGTCTTTGAGGCGATCTTCGATGCGGGGTCCATAAAATTCGCTGCAGGGATGATTGGGCGTGCACTGTTGTAACGCGTATGTCTGACGCTGCATTTGAACCACACGAAGGGGGTCTGCGGCAGGTCGTGGGATGAGCTGAGTGGAGCTTCACTCATGCTGTACGGCGCCGGGACGGAGAGGTGGTGGACATGTTGGTGGCCAGAGAGAGCCCCCCTGCAAATCAAGGAATTC**GTGATTG

Lmex.08_29:190,165..190,764

CCAACC**CCCTGATGCTGGGGGGATACCTTAGCGTGGTATCTCAGGGTCCAGTACACCCCCACTCTCTGTGTGGGGAAGCCAAGCAGCCCCTCCCCCTATCCCTGCCAACGCCGAGCCACTTCTCGTGGTGACAGGGTCAAGCACCTTCGACATGGCGGGAGGTCAGAGCGATGCATCGCTGCTAGTTTCGGTGGTGAGGTCCTGGATTGGGGTTGTGTTGAAGCCGCCTGCGACAGTGAGCACGCTTGTACCATTCACATGATAGGCGATGTGTCCGCGTGGCATGAGCGTCCCTTACCCGAGCCTCACTGCCTGATGGCGGAGGCGGCCTGCGCGATCGCACGGGAGATGCACCCGGTGGCGACCGGTATGATGGGGGCTACTGTAAGACGGTCTTCGATGCGGGACGAGCAGTATTCGTTGCAGAGATGACTGATTCTACATTGTTGTAAAGCGTGTGTCTAGCGCTGCATTGCACCACACGATGGGGCCTGCGGCAGGCCGTGGGGTGGGTTGCGTGAGGTTTCACTCATGTTGTATGGCAGAGAGAGGGGGAGAGAGATGGGCACGCTGGTGATGAGAGGTAAAAAAAA**TGACCGA

Ld20_v01s1:1,036,612..1,037,195

CCAACC**CCCTGATGGCGGGGGGACACCTCAGCGTGGTATCAGGGTCCAGTACCCACTCTGTGTGGGGAAGCCAAGCAGCCCCCCTATCCCGGTCAATGCATAACCACTTCCGGTGGTGGCAGGACCATGTACCTGCGACGTGGGGGGGGGAGATTAGGGCGATGCATCGCTGCTAATGTCGCCTTTGTGGTCCTGGGCGACTTTGCGTCGGAGCCACCCGCGACAGTGACCACGCTTGTACTACTCACATGATAGGCGACGTGCCCGCATGACGGGAGCGTATCTTACGCGGGCCTCACTGCCTGATGGCGGAGGTAGCCTGCGCGACCGCGCGGAGGATGCACCAGGTGACGATCTGCATGATGGGAGCTACTGTGCGGCGATCTTCGGTGCAGGGCGAATAGTATTCGTTGCAGGGATGACTGGGTCTGCATTCCTGTAACGCGAGTGTCTACCGCTGCATTGCACCACACGATGGGGCCTGCGGCAGGCCGTGGGGCGGGTTGAGTGGAATTTCACTCATGTTGTACGGCAGAGAGAGAGATGGACACGCTGGTGGTAAGAGGTAAAAAAAAAA**TGACTGA

KB453339:66,838..67,364

CCAACC**CCCTGATGATGAGTCACCTCTCAGTGCCTGACCTCCAGGGCTTAGTGCCCCCGCTCTATGTGGGGAAGCCAACCAGTCCCCCTTCATCCCTACCTCCACTGAACCACTCTAGGCCGTGACGTGGTCCAGTACCCGCGACACGGGGAGGTCAAGGCGCGGTGCATCACTGCGGATGCCGGCGGTGTAATGTCCTGGACGGCGTGGCGTCGGAGCGACCGGCGACAGCGGGCACGCCTCTGCCGCGTGCATGATGGGCGGGGTGTCAGCGCGGCTCGAATGCATCTCGCGCGGCCCTCGCTGCTTGCGGGTGCGTGGGGCCTGGGCCACGCCGTGGGTCGCACCACGTGACGGCCGGCCTTGTAGGGGCGGCTGCACGGCGACGTGCGGAGCGTTGGTGGGTGGGCAGCGCTTGGGGCGGAGGGCGGCCGTGCGGAGATGGCTGTGTCGGCGCATTGGAGGACCGCGTATCTAGTTTGCTGTGTCGCACGACGCGATGGGTCTGTGGGACACGNNNNNNNNNN**

*Trypanosoma brucei* SIDER1 sequences:

Tb927_10_v5:452,220..452,812

AATTTATACTGT**CCCTTATGTTAGAGGTAGAAGCCTCATTGTGGTGTCAGGGTCTAGTACGCAGGAATAATTAAATTTTCTATGGAAGCTAACTGTCACCACATGAACACTGGAATATCGACCACAGTGGGCAGCTGCTGGGGTGCCATTGTGAGTGCACTTTTGGACGTTGAAGATATTTATGTGTGTGAAGCGCAAATCAACACGCGGATATGTGGCTACTTCATGTTTTACAGTTGAGCACCTTTGCAAGTAGAACCCATCGAAGTCGTATACGTGCCATTAGCTGTAGAGCTAGTGGTGAATTTACAGGGTGAGATGCCACAACAGCTATAGGAGGGGTAGAAGGCTACAATGAAGACCATCCCTTTATACGACCGTGCGAATCGTGGTCTCCATGCACTGGCAAAGTGGGTGGGTAATAATCTCCACTTAAGAGGGTTTTAGTAGGCAGTGGCTCGTTCTCCAAGGCTGAGTCCCACACTATCATGTCATGTGACATGATCTGCATAAACGCATTTCCTTCTGTTCGCTAGTGTGGCGTGGCCACGATTGTCGTCAATCCGCACATTGAAGAAAAAAA**CTTATGCTGT

Tb427_10_v5:452,323..452,915 (TbSIDER1α)

AATTTATACTGT**CCCTTATGTTAGAGGTAGAAGCCTCATTGTGGTGTCAGGGTCTAGTACGCAGGAATAATTAAATTTTCTACGGAAGCTAACTGTCACCACATGAACACTGGAATATCGACCACAGTGGCCAGCTGCTGGGGTGCCATTGTGAGTGCACTTTTGGACGTTGAAGATATTTATGTGTGTGAAGCGCAAATCAACACGCGGATATGTGGCCGCTTCATGTTTTACAGTTGAGTACCTTTGCAAGTAGAACCCATCGAAGTCGTATACGTGCCATTAGCTGTAGAGCCAGTGGTGAATTTACAGGGTGAGATGCCACAACAGCTATAGGAGGGGTAGAAGGCTACAATGAAGACCATCCCTTTATACGACCGTGCGAATCGTGGTCTCCATGCACTGGCAAAGTGGGTGGGTAATAATCTCCACTTAAGAGGGTTTTAGTAGGCAGTGGCTCGTTCTCCAAGGCTGAGTCCCACACTATCATGTCATGTGACATGATCTGCATAAACGCATTTCCTTCTGCTCGCTAGTGTGGCGTGGCCACGATTGTCGTCAATCCGCACATTGAAGAAAAAAA**CTTATGCTGT

Tbg972_10:401,727..402,319

AATTTATACTGT**CCCTTATGTTAGAGGTAGAAGCCTCATTGTGGTGTCAGGGTCTAGTACGCAGGAATAATTAAATTTTCTACGGAAGCTAACTGTCACCACATGAACACTGGAATATCGACCACAGTGGCCAGCTGCTGGGGTGCCATTGTGAGTGCACTTTTGGACGTTGAAGATATTTATGTGTGTGAAGCGCAAATCAACACGCGGATATGTGGCCGCTTCATGTTTTACAGTTGAGTACCTTTGCAAGTAGAACCCATCGAAGTCGTATACGTGCCATTAGCTGTAGAGCTAGTGGTGAATTTACAGGGTGAGATGCCACAACAGCTATAGGAGGGGTAGAAGGCTACAATGAAGACCATCCCTTTATACGACCGTGCGAATCGTGGTCTCCATGCACTGGCAAAGTGGGTGGGTAATAATCTCCACTTAAGAGGGTTTTAGTAGGCAGTGGCTCGTTCTCCAAGGCTGAGTCCCACACTATCATGTCATGTGACATGATCTGCATAAACGCATTTCCTTCTGCTCGCTAGTGTGGCGTGGCCACGATTGTCGTCAATCCGCACATTGAAGAAAAAAA**CTTATGCTGT

Tb427_09_v4:3,011,406..3,012,022

CTATTTCAAGCACCGTCAAC**CCCTTGGCGTTAATGGTAGCAGCCTCATCGTGGTGTAAGGGTCTAGTACCCAGAAATAATTAAATTTTTTCAGAGGAAGCCAACCGTTACCACATGAAAACTGGCATATTGGCCACAGTGAGTGATTTCTGTGGCGCCAGGGCAAGTGCACTTTTGGACATTGAAGATGTTTATGTGTGCGCAGCGCAAATCGATCCGCGAAAAAGTGAATACTTCATGTTTTGGGGTTAAGTACCCTGTCCAAGGGAAACCCATCGAAGTCGTCTTTATACCACTAGCTGTAGTGCTATCGGATGACTTCAGAGGGTTAGATGCCACAAAAGCTATACTGTTGGTAGTGTGCTGAAATCAACATCGTCCCTTTATATGGCCAACGAATCATGGTCACGATCCACTGGCAAAGTGTGTGGGTAATAATCTCCACCAAAGGAGGTTTTAGAAGGTAGCGGCTTGCCATCCAAGGCTGAGTCCCACACTATCATGTCTTGTCACATTATGTGCATAAACGCATTTCCTCCTATTTCCTATTATGCGGTGACCACAACCGTCGTCAACCCGTACCATGAAGGAAAAAA**TGCACGTATACCACTTCTTTTT

Tb927_09_v4:3,010,329..3,010,946

TTCTATTTCAAGCACCGTCAAC**CCCTTGGCGTTAATGGTAGCAGCCTCATCGTGGTGTAAGGGTCTAGTACCCAGAAATAATTAAATTTTTTCAGAGGAAGCCAACCGTTACCACATGAAAACTGGCATATTGGCCACAGTGAGTGATTTCTGTGGCGCCAGGGCAAGTGCACTTTTGGACATTGAAGATGTTTATGTGTGCGCAGCGCAAATCGATCCGCGAAAAAGTGAATATTCATGTTTTGGGGTTAAGTACCCTGTCCAAGGGAAACCCATCGAAGTCGTCTTTATACCACTAGCTGTAGTGCTATCGGATGACTTCAGAGGGTTAGATGCCACAAAAGCTATACTGTTGGTAGTGTGCTGAAATCAACATCGTCCCTTTATATGGCCAACGAATCATGGTCACGATCCACTGGCAAAGTGTGTGGGTAATAATCTCCACCAAAGGAGGTTTTAGAAGGTAGCGGCTTGCCATCCAAGGCTGAGTCCCACACTATCATGTCTTGTCACATTATGTGCATAAACGCATTTCCTCCTATTTCCTATTATGCGGTGACCACAACCGTCGTCAACCCGTACCATGAAGGAAAAAA**TGCACGTATACCACTTCTTTTT

Tb927_09_v4:2,520,024..2,520,638

TCTATTCCAAGCACCGTCAACC**CCCTGGCGTTAGTGGTAGCAACCTCATTGTGGTGTCAGGGTCTTGTACCGAGGAAGAATAAAAATTTTTTGCGGAGGCTAACTGTTACGACGTGAAAACTGGTATATCTACCGGGGTAAGTGATTGCTGTGGTGCCATAGTGAGTGCACTTTTGGACGTTGAAGATATTTATTTGTGTGAAGCGCAACTCAATCTGAGAAAAAGTGAATACTTCATGCTTTGGGGTTAAGTACCCTGTCCAAGGGAAACCCATCGAAGTTGTATACGTGTCATTAGCTGTAGAGCAAGTGGTAAATTTACAGGGTGAGATGCCGCACCAGCTATATGTGGTGCCGCGTCCTGCAATGGAGACCATGTATATAGAACGGTGGCCGTGTTGTGTTTACCATCTGTTTGCGAAGTTGGTTGGTAATAATCTCCACATGAGGAGGCTTTGGTGGATAGTGGCTTGTTCTCCAAGGCTGAATCTCACACTATCACGCCATGTTATATGATGTGCATAGACACATTTCTTCCTCATTACTAGTGTAAGGTAGCCACGATAGCCGTGAATCCGCACAGTGATAAAAAA**GTCCATGTATATCACTTCTTTC

Tb927_11_01_v4:4,592,751..4,593,343 (TbSIDER1β)

CTATTCCAAGCACAGTCAACC**CCCTGACGTTAGTGGTAGCAACCTCATTGTGGTGTCAGGGTCTTGTACCGAGGAAGAATAAAAATTTTTTGCGGAGGCTAACTGTTACGACGTGAACACTGGTATATCTACCGGGGTAAGTGATTCCTGTGGTGCCATAGTGAGTGCACTTTTGGACGTTGAAGATATTTATGTGTGTGAACCGCAACTCAATCCGAGAAAAAGTGAATACTTCATGCTTTGGGGTTAAGTACCCTGTCCAAGGGAAACCCATCGAAGTCGTATACATGTCATTAGCTGTAGAGCTAGTGGTGAATTTACAGGGTGAGATGCCGCAGCAGCTATATGTGGTGCCGCGTCCTGCAATGGAGACCATGTATATAGAACGGTGGCCGCGTTGTGTTTACCGTCTGTTTGCGAAGTGGGTTGGTAATAATCTCCACATAAGGAGGCTTTGGTGGGTAGTGGCTTGTTCTCCAAGACTGGACCTCACACTATCACGCCATGTTATATGATGTGCATAGACACATTTCCTCCTCATTACTAGTGTAAGGTAGGCACAATAGCCGTGAATCCGCACAGTGATAAAAAAA**GTGCACGTATATCACTTCTTT

Tb427_11_01_v4:4,593,961..4,594,573

CTATTCCAAGCACCGTCAACC**CCCTGGCGTTAGTGGTAGCAACCTCATTGTGGTGTCAGGGTCTTGTACCGAGGAAGAATAAAAATTTTTTGCGGAGGCTAACTGTTACGACGTGAAAACTGGTATATCTACCGGGGTAAGTGATTGCTGTGGTGCCATAGTGAGTGCACTTTTGGACGTTGAAGATATTTATTTGTGTGAAGCGCAACTCAATCTGAGAAAAAGTGAATACTTCATGCTTTGGGGTTAAGTACCCTGTCCAAGGGAAACCCATCGAAGTCGTATACGTGTCATTAGCTGTAGAGCTAGTGGTGAATTTACAGGGTGAGATGCCGCACCAGCTATATGTGGTGCCGCGTCCTGCAATGGAGACCATGTATATAGAACGGTGGCCGTGTTGTGTTTACCATCTGTTTGCGAAGTTGGTTGGTAATAATCTCCACATGAGGAGGCTTTGGTGGATAGTGGCTTGTTCTCCAAGGCTGAATCTCACACTATCACGCCATGTTATATGATGTGCATAGACACATTTCTTCCTCATTACTAGTGTAAGGTAGCCACGATAGCCGTGAATCCGCACAGTGATAAAAAA**GTCCATGTATATCACTTCTTT

*Trypanosoma vivax* SIDER1 sequences:

TvY489_03:1,238,673..1,239,284 (TvSIDER1)

AATGAAGGTAATA**CCTTGATGTAAGTGGTAAAACGCCTCATCGTGGCATCAAGGTCTAGTACCTGCGAAATTCCTTTGGAGTTCGGAGGGAAGCTAAGTTTTACCACGCTCAAAACACTGGCCTATCGACCATGGAAATTGCTTCTATGGGGCCAGGGCGCATGCAGTTGATGCCTTTATGGAGAAAAACGTGACTGCGCAAATCGATCCGCGGAGATGTGGCTACTATGTGTTCCGCGGTCTAGTACCCTGTCTAAGGGGAATCCACCGAAGTCGTGTACATGCCGCCAGCTGTAGAGCCGGGGGTGACTTCAGAGGGTGAAACGCCACAGCAACTATGGAGGGGTAGCGGGTTGCATTGGAGACCGTCCCTCCAGACCAACGTGCGAGTCGTGGTCACCATCCACTGGCGAGGTGGGCGGGAAATTAATCTCCACCCAAAGGGGGTTTTAGTGGGTATTGGCTATTTCTCCAGGTTTGAGTCCCACACTACCATGTTTAGTGGCGTGGTGTGCGTAAATGCATTTCCTCCTCTTCGCTAGTGTTGGGTGACTACTTCGGTCGTTAATCCGCACAATGAAGAAAAAAAAAAAAAAAAAAA**TGAAGGTAATA

TvY486_bin:11,221,740..11,222,352

AATAAATAGGTAA**CCTTGATGTAGGTGGTAAAACGCCTCATCGCGGCGTCAAGGTCTAGTACCTGCGAAATTCTCTTGGAGTTTGAAGGGAAGCTAAGTTTTGCCACGCTCAAAACGCTGGCCTATCGACTGTGGGAATTGCTCCTATGGGGCCAGGGCGCACGAAGTTAATACCTTATGGAGAAAAACGTGACTGCGCAAATCGATCCGCGAAGATGTGGCTACTATGCGTTCCGCGGCCTAGTGCCCTGCCTAAGGGGAACCCGCCGAAGTCGTGTACATGCCGCTAGCTGTAGAGCCGGGGGTGGCTTCAGAGGGTGAAACGCCACAGCAACTATGCAGGGGTAGCGGGTTGCAATGGAGACCAGCCCTCCAGACCAACGTACGAGTCGTGGTCACCATCCACTGGCGAGGTGGGCGGGAAATTAATCTCCACCCAAAAGGGGTTTTAGTGGGTATTGGCCATCTCTCCAAGTATGAGCCCCACGCTACCACGCTTAGCGGCGTGGTGTGCGTAGATGCATTTCCTCCTCGTCGCTAGTGTGGGGTGACTACCTCGGTCGTTAATCTGCACAATGAAGAAAAAAATAAATAAATAGA**TAAAATATGACAA

*Trypanosoma congolense* SIDER1 sequences:

T.congo.pschr.1:110,891..111,480

AAGCCTTGAGAAA**CCCTGATGTTAGTGGTAAAAGCTTCATCGTGGCATTATGGTATAGTACCCAGAAATGCTTATTTCACAGGGAAGCCAAGATTTACCACGCAAGAACATTGGCTTATGGACAGTGGGAGCCACTTCCATGGAATCAGGGCTCACGGAATTCGTACTTTTTGTGGAAACGAATGCTTTAGCGCAAGCCGATCCACCGAGATGTGGCTGTTGCGTTCCATGGTTTAGTACCCTGTTTAAGGGAACCCACCGAAGTTGCATACATGCCGCTGGCCATAAGGTAAGTGGTGACTTCAAAGGGTGAAATGCCATAGCGACTATGGATGAGTAGCGGGTTGCAATGGAAACAATCCTCCCGGATCAATGTGCAAGCCGTGGTCACCATCCACTGGCGGAGTGGGCGGGAAATCATCTCCACCCAAAGGGTGTTTTAATGGGTAGCGTCCTTGTTCTCCAAGGCTGAGTCCCACACTACTACGTTGTGAGGCGTGGTGTGCGTAAATGCATTATTTTCTCTTCGCTAGTGTAGGGTGGCAACTTCGGTCGTTAATCCGCACGATGAAGAAAAA**GGGCCTTGGAAA

T.congo.pschr.3:45,521..46,110

AAGCCTTGAGAAA**CCCTGATGTTAGTGGTAAAAGCTTCATCGTGGCATTATGGTATAGTACCCAGAAATGCTTATTTCACAGGGAAGCCAAGATTTACCACGCAAAAACACTGTCTTATGGGCAATGAGAGCCACTTCCACGGAATCGGGGCTCACGGAATTCGTACTTTTTGTGGAAACGAATGCTTTAGCGCAAGCCGATCCACCGAGATGTGGCTGTTGAGTTCCGCGGTTTAGTACCCTGTTTAAGGGGAGCCCGCCGAAGTCGTGTGCATGCTGCGAGCCATAAGGCCAGGGATGGCTTCAGAGAATGAAATGCCACAGCAACTATTTAGGGGTAGCGGGTTGCAGTGGAGACCGTTCCTCCGGACAAATGTGCAAGTCGTGGTCACCATTCACTGGCGAAGTGGGCGGGAAATCATCTCCACCCAAAAGGGGTTTTAGTGGGTATTGACTATTTTTCCAAGTCTGAGTCTCACACTACTACGCTGTGAAGCGTGGTGTGCGTAAATGCACTATTTTTTCTTCACTAGTGTAGGGTGGCAACTTCGGTCGTTAATCTGCACGATGAAGAAAAAA**GGCCTTGGAAA

T.congo.pschr.1:117,249..117,838

AAGCCTTGAGAAA**CCCTGATGTTAGTGGTAAAAGCTTCATCGTGGCATTATGGTATAGTACCCAGAAATGCTTATTTCACAGGGAAGCCAAGATTTACCACGCAAGAACATTGGCTTATGGACAGTGGGAGCCACTTCCATGGAATCAGGGCTCACGGAATTCGTACTTTTTGTGGAAACGAATGCTTTAGCGCAAGCCGATCCACCGAGATGTGGCTGTTGCGTTCCGTGGTTTAGTACCCTGTTTAAGGGAACCCACCGAAGTTGCATACATGCCGCTGGCCATAAGGTAAGTGGTGACTTCAAAGGGTGAAATGCCATAGCGACTATGGATGGGTAGCGGGTTGCAATGGAAACAATCCTCCCGGACCAATGTGCAAGCCGTGGTCACCATCCACTGGCGGAGTGGGCGGGAAATCATCTCCACCCAAAGGGTGTTTTAATGGGTAGCGTCCTTGTTCTCCAAGGCTGAGTCCCACACTACTACGTTGTGAGGCGTGGTGTGCGTAAATGCATTATTTTCTCTTCGCTAGTGTTGGGTGACTACTTCGGTCGTTAATCCGCACGATGAAGAAAAAA**GGTCTTGGAAA

T.congo.pschr.11:3,750,506..3,751,107

AGAGGAATTCC**CCCCGATGTTGGTTGCAAACGCCTCGTCGTGGCGGCAGGGCAAAGTACCTAGAAATACTTGTTTCACAGGGAAGCTAAGATTTACCACGCAAAAACACTGGCATATCGACCATGGGAGCTGCTTCTATGGAACCAGGGCGCACGGAATTCGTACCTTTTGTGGAAACGAATACTTTAGCGCAACCCGATCCGCGGAGATGTGGCTAATTTGTGTTCTGCGGTTGAGGACCCTGTCTGAGGGAAACCCACCGAAGTCGTGCACATACCGCGAGTCTGAAGAAGGTAAGTGACGACTTCAAAGGGTGAAACGCCACAGTAACTATGGAGGGGTAGCGGGTTGCAATGCAGACCGTCATTCCGGACCAATGTGCGAACCGTGGTCACCATCCACTGGCGAAGTCGGCGGGAAATCACCTCCACCGAAGAGAGTTTTAGTGGGTGGTAGCCTCGTCCTCCAAGGCTGAGTCCCACACCACCGCGTTGTGAGGCGTCGTGTGCGTAAATGCATTATTTCCTCCTCTTCAACGGTGTAGAGTGACTACTTCGGTCGTTAATATGCAGGATGAAGGGGAAAAAA**GATTTGGGAATTAA

T.congo.pschr.10:2,982,978..2.983,564

TAGGAAGGATAT**CCCTGATGCTAGTGGTAAGTGCTTCATCGTGGCGTCAGTGTCTAGTGCCTAGAAATGCTTATCTCACAGAGGAGCCAATATTTTCCACACAAAAACACTGGCCTATCGACCATGGGAGCTACTTCTATTGAGCCAGGGCGCAAGGGATTCGCATCCCTTATTAAGAGTGATACCGCACAAACCGATCCGCGGAGATGTGGCTGCAATGTGTTCTGCGGTTTAGCATCCTGTCTCAAGGGAGCCCACCGAAGTCGTACACTTGCCGCCAACCATGAGGTAAGGGGCGATTTCAAAGGGCGAAACGCCACAGCAATTATCAAGTTGTAGCGGCTTGCAGTGGAGACCGTACCCCCGGACAAACGTGCGAGTCATGGTCACCATCCACTGGCGGAGTGGGCGGGAAATCACCCCCACCCAAGGGGTTTAGTGGGTAATGGATCGGTCTCCAAGACTGAATCCCACATTACTATGTTGTGAGGCGTGGTGTACGTCAATGCATTATCTCCTCTTCACCGATGTAAGGTGGCTACTTCGGTCGTCAATCCGCACAGTGAAGAAAAAAA**TAGGAAGGATAC

T.congo.pschr.10:400,469..401,062 (TcoSIDER1α)

ACCATCACCACT**CCTTGATGTAGGTGGTAAACGCCTCATCGCGGCGCCAGGGTCTGGTACCTATGAAACTCAGTTAGAGTTAGTAGGGAAGCTAAGCTTTACCACGTAAAAACATTGGCCTATTGACCATGGGAGATGCTTCTATGGAGCCAGGGCGCACGAAGTTGATGCCTTTATGGAGAAGTGTGAAACTGCGCAAATCGATCCGCTGAGATGTGCCTACTACGTGTTCCGCGGTCTAGTACCCTGTCCAAGGGGAACCCACCGAAGTCGTGTACATGCCGCTGGCCATAAGGTAAGTGGTGACTTCAAAGGGTGAAACGCCACAGCAACTATGGAAGGGTAGCGGGTTGCAAAGGCCACCGTCCCTCCAGACCAACGTGCGAGTCGTGGTCACCATCCACTGGCGAGGTGGGCGGGAAATTAGTCTCCACCCAAAGGGGGCTTTAGTGGGTATTGACTGTTTCTCCAAGTATGAGTCCCACACTATCACGTTATGGGGCGTGGTGTGCGTAAATGCATCACTCCTTCGCTAGTGTTGGGTGACTACTTCGGTCGTTAATCCGCACAATAAAGAAAAAAAA**CACCCCTCCT

T.congo.pschr.5:603,310..603,918 (TcoSIDER1β)

CTTTGAATGTACAGCAGCATGG**CCCTGATGTTAGTGGTAGATGCCTCATCGTGGCGTCAGGGTGTTGCGGCTTGGGATGCTTAATTCACAGGGAATCTGACATTTACCACGCAAGAACATTGGCTTATCGACCATGGGAACCACTTCTATGGAATCAGGGCTCACGGAAATCGTACCTTTTACGGGGAAGAATGATACTGCGCAAATCGATCCGCAGAGATGTGGGTATTATGTGTTCTTTGGTTGAGGACGCTGTCCAAGGGGAACCCACCGAAGTCGTGTACATGCCGCCAGCCGTAGGGCCTGGGGTGACTTCAAAAGGCTAGACATCACAGCAACTGTGGAGGGATAGCGGGTTGCAATGGAGACCGTTCTTCTGGACCAAAGCTCAAGTCATGGTCACCATCCACTGGCGAAGTGGGCGGGAAATAATCTTCACCCAAAGGGGGTTTTAGCGGGTAGCGGCCTAGCACTCCAAGGCTGAGCTACACACTGTCACGTTGTGAAGCATGATGTGCGTAAATGCATTTCCCCCTCTTCAACGGTGTAGAGTGACTACTCCGATTGCTAATCCGCAGAATGAAAAA**GGAAAATAGCATAGCGTCAAAG

T.congo_bin:13,361,355..13,381,926

**CCCTGGTGACAGTGGTAAACGCCTCATCGCGGCGTCAGGGTCTAGTACCTATGAAACTCATCTAGAGTTAGTAGGGAAGCTAAGCTTTACCACGTAAAAACATTGGCATATCGACCATGGGAGATGTTTCTATGGAGCCAGGGCGCAAGGGATTGATGCTTTTATGGAGAAGTATCAAACTGCGCGAATCGATCCGCGGAGATGTGGCTACTACGTGTTTCGCGGTCTAGTACCCTGTCCAAGGGGAACCCACAGAAGTCGTGTACATGCCGCTAGCTGTAGGGTTAGGGGTGACTTCAAAAGGTGAAACGCCACAGCAACTATGGAGGGGTAGCGGGTTGCAATGGAGACCGTCCCTCCAGACCAACGTGCGAGTCGTGGTCACCATCCACTGGCGAGGTGGGCGGGAAATTAATCTCCACCCAAAGGGGGTTTTAGTGGGTATTGACTATTTCTCCAAGTCTGAGTCCCACACTACCACGCTTTGCGGCGTGGTGTGCGTAAATGCATTTCCTCCTCTTCGCTAGTGTTGGGTGACTACTTCGGTCGTTAATCCGCACAATGAAGAAAAA**

T.congo_bin:7,431,359..7,431,923

**CCCTGATGTTAGTGGTAGATGCCTCATCGTGGCGTCAGGGTGTTGCGGCTTGGGATGCTTAATTCACAGGGAATCTGACATTTACCACGCAAGAACATTGGCTTATCGACCATGGGAACCACTTCTATGGAATCAGGGCTCACGGAAATCGTACCTTTTACGGGGAAGAATGATACTGCGCAAATCGATCCGCAGAGATGTGGGTATTATGTGTTCTTTGGTTGAGGACGCTGTCCAAGGGGAACCCACCGAAGTCGTGTACATGCCGCCAGCCGTAGGGCCTGGGGTGACTTCAAAAGGCTAGACATCACAGCAACTGTGGAGGGATAGCGGGTTGCAATGGAGACCGTTCTTCTGGACCAAAGCTCAAGTCATGGTCACCATCCACTGGCGAAGTGGGCGGGAAATAATCTTCACCCAAAGGGGGTTTTAGCGGGTAGCGGCCTAGCACTCCAAGGCTGAGCTACACACTGTCACGTTGTGAAGCATGATGTGCGTAAATGCATTTCCCCCTCTTCAACGGTGTAGAGTGACTACTCCGATTGCTAATCCGCAGAATGAAAAA**

*Trypanosoma congolense* L1*Tco* sequences:

T.congo.pschr.10:560,019..564,770

AACCTTCATATA**CTCTGGCGCAGCCGGCCACCTCAACGTGGTGCCAGGGTCCAGTACTCTTCATTGGAGAGGAAGCTAAGTGCCAGCTACGCTCTCGATGCTATCGTTTGCGAAGTTGGCTCCCGGCTAAAGGGCCGGAAGGGTGTATATTGAAGCCTAGCTGTAGTCCACCCGTGCGAGCTGTATTGGTCACAACCCTGTCAAATGTCATGAGCGCTCACCTGCGCCATCCCAACCGACAATGTGCCTTGCAAGAAGCACTGCGTCAGAAGTTGCTGTTGTGTGGTGATGTGGAAGCAAATCCTGGACCTCTTACAACACTTGAACTAAACGTTCAGTCGCCGTCAGTGGCTAAACCTTTTTCTCTGCTGCCCAGGGGGTCGGATATTATTACACTGCAACAGACTTGGAAATCGGCAAAGGAAATTTCGTCTTTGAAGACACATCCTTATATTACGTACTCTTACCCACGTAGAGGACGAGGCGGCGGAGTTGCCATTATGGTAAGGAATACACTGAAGTCAAGACAAGTTACTATGAAAATTCCTGTATATGATACGAATACCGAAGCGGTGCTCGTGGAAGTAGTATTACGAAACGGTAGTAATTTGTACATCGCTAGTGTCTATCTTCCTCCACCAGCAGCTGTCACGTCAACTTTGCACAAGCTTGTGGCTACTGTGCCTGCGTCTTCTCCCTTTCTCCTATGTGGTGACTTCAATTTACATCACCCATTGTGGGCATTAGAGGGGGATGAAGCTCCGTCGGATACAACACAAAAGCTCTTGGACATCACTCTTGGAGCTAAACTCACCCTCGCAAACGAGCCGGGTTTTACGTTCGCTAGAGGTCCTACTGAGTGCTCGTGCACTGACCTGACCTTTCAAAGGTTTTTAACTGTTGAATGTTGGACAGCGCGTGTTACCATCCACAGTGATCATTTTTTGATAAAGTTTTCCGTGAGGGCTTCCCACCGCAATGAGATCCCCCCAGCGGCGCATGTTAGGCGAAGACATTTCTACAGCTGGAAAAAGTGTGACTGGGATTCTTTTCGGAATAAAATGGATTCTCAGCTCCCTAATTTCGATCCTAGAAACATTCATCGGAACATAAAGGCCTTTACGGACTGTTATTATACCGCACTAAGACGACACTTCCCGCGCGGTATGATAAAGGATGGTCCCATTTTCTGGGACCGTGAGATAATAGAAGCTGAACGTTACGTGGAGACACTGAAATCAATATACATGAATATGCCATCTTCAGCGCAATTGGCGGCGCTGAATGAAGCTAAGGATAAATATATGGACACTGTCAGGCAAAGACTGAACACTACTTTTCGTCACCGGCTGGGGAAATTGTCCCCTGGAGAACACCTCTCCTGGAAGTACATCTCGTCACAGAATAGGGCTACCACCCCTCTTGCAGACTCCCTTCTTTTAAGTGTAGGACACAAGCAGCTTAGTACCTCGCGTCACATTGCGAACGCATTCAATCGCAATTTTTTCCCCCTCTCAAGGTAGGTGGAGATTACTTAAGTTTGCTTCCGGAAGGTCTTTTAAAAGAGGTAAGGAAAGTTCGCCTCTGTCTTCTTCTGCTCTTTCTCTAGGTTTTTCACCTCATTTCTCTTCTCTTCCCTCACTAGGTACACACATAGGGCTTCCTTTTGATGCTGCTTCTTTCCCCACCCTCTCCTCTCTAACTGCACTCTCCTTTCTTGACGCCCCATTCCGCCCGGCGGAGCTACTTTCAGCCCTGAGGAATACCCCTTGTGGAAAAGCCCCCGGCCTGGATGAGATTTATGCTGAGACATTTGGACATTTTTCTGAAAAGACAGTGAGATATCTTCTGAGGTGCATTAATCAGAGCTGACTGTCAGGAATTATCCCCGTTCAGTGGAAACGTGCAACTGTTATCCCACTGCTCAAGCTTGGAAAACGGCCGGATGATACCAACTCGTATAGGTCTATTGGCCTTACGTCGGTTATACGTGAGGTAGCTGAGAGAATGGTTCTGCGACGGTTATTGTGGGTATGGACTCCCCATGCCCACCAGTACGCGGACAGGAAAATGCATACGACGACAATGTAATTGGCGCAATTAGTAGATACTGTTGAGCATAACCGAAATCACTATTTCGATGTCCGTCTCCCTAAAAAGAGCGGCATTGGCGACCAAGTGCACTACAGACCGCATTGCACATTGTTGGTTCTCATTGATTTCAGTCAAGCCTTTGACTCAATCGACCACCATGTGCTGAGCAGGAGGCTCGCACTTATTCCAGGGGTTTATTGTAAGAGGTGGCTTCGAAACTTACTATGTGATCGACTTGCGCGGAACAGGGTCGGTAATCGCAAGAGTGCTCAAAGACCGGTGCTAAGAGGAGTTCCTCAGGGCTCCGTAGTGGGACCATGCCTGTTTTCCCTCCGCGTACACCCGCTCCTCAACTTGCTGAATACGGACCCGGAGATATCGGCCGATATGTATGCTGATGACTTATCCATTACCATAAAGGGCCGATCTCGTGAAGAGGCCGTCCTGCAGGCAAATTCTTATCTGGTTAAACTGCATCGATGGACATCAGAAAATGGTCTACAGGTGAACCCGTTAAAGTGTGAAGCCGCGTGGTTTACGATATCAACACACACTGAAGATGATAAGGACCGTGAAGGGAGGTTTCCACTTCTCTTCAATGGTCATGAGATACCCATCTCGACTATGGGATCCACACACTTGCCAAAGCTGCTGGGAGGACCCCTAGATACACGTATGAATTTCAATTCCGCTGCTACTTCTCGATGTACAGCCACTTCGACCCGAATCGCACAACTGAAAAGTGTGGCGCACAAAAAGGCTGGTCCGCTTCCGCATGACATGCGTACTTTCGTCATTGGTTACGGAGCGTCCAAGCTCTTATATGGAAGTGAGATGATTTGGGCGTTAGCTGACGACTCAGCAAAGAATGCGATGATGAGGACATATGCGAACATAGACAGAATAGTAAGTGGTGCATTGTCGACGACTGACCCGGAATCTGCGCTCCTGGAGGCGAATATGACGCCGTTACACATTCTTGCATTAAGGGCGCGCTTTGCTTTATTTGAGCGCGTTCGATCATGCCAAAAAGAATGGATTCGGCGTCCGCCTCCAGAGCCCCCCGTGTAAAGGTTTTCGCATATCACCGATATCGCGAGAGGCGGTGTATTCACTAGTCGGTGATTTAACCGAAGAATATGGAGTCAACCGAAACAGCGTTAGGGAAAGGAGATTCTTCAAGTCTGCTGTTCCTCCATGGTCGGTTTCCCAAGCAAATAAAGTGACGTTTGGGCTTACTGTGGAATATGACAAATCTCTCACACACAAAGATGCAATCCGCTTAGCAAAGAAATGGGCCAGTCTACATGAGATTGGTAAGCATAACCATTTTCAGTGGCTGATTGCAACAGATGGTGGTATTCAATCACCCATGTCGGCCGGTGTCGGGCTATTGTTTAAATCTGTCTCGCATCCCGTACTGATGAAACAAGTCAGCGTGAACTGTGGATCCGTATCAAGCAGTTACAGGGCAGAGTCCGTGGCGATGCTATTGGCTCTGGATAGGTTGGTTATGCCAATGGCGGATGTCAAACATAAGACGTTACTCATTGTCACAGATAGCCAATCCCTCCTGAATGCATTAAGCAAAGGTCCGCTAAGTCAATGTGACTACACGGAGGATGTGATATGGACTAGACTTATTGAGCTCACACTGCAAGGATGGTTAATTCACTTTCAATTCTGTCACAGTCATTGTGGAGTAGTAGTGAATGAGATGGCAGACGAATATGCAACTCAATGCATGGAGAATGGTCACTTCACTGAACTCTCAGTCAAACCACTGTGGCACAAAGATCTTGAGGCTCTCATCACCAGACAACTCAAAAAACGGTGGCTCGCCTCGTTAAGAGTCGACACGTATCGCTACAAGCTGTGTGGAGCGAAACCGTCAGACCTTAGTGGACTGGACCTGATTGATGGTACAAAGTTAACCAGATCAGAAATAGTTGGGTTGGCTAGAGCTCGTTGTGGAGAATCAGAATATTTCGGACGTTTATTTTGGAGTCTGAGGGACTGCTTGCCGACATGCAGGTTGTGCAACTGCACGCCAGAACAGGCCGCTGTACTGTCACACTCTTCTCTTCCAGGAGAGGGCCTGGATGCATCCACCAACACAACACAAGAAACGGCGGAGAGGCCAAGGGCAAATAAGAATCGGAGACGAGAACCATGCCCATACTGTGATGCTGTCTTTGTTGGATTTACAAAATTAAAACAACACTGTAAAACACAGCATAGTGATCAGACAAAACCCGCCGAGCAACTTCAATGTGATTTTTGTGGTGAGGAGTACTCCAACAGGAGGAGTACCGCGCAGCACAGAATGAGGTGTAAACAAAATCCAAACTACATCCGGCTAAATAACAGTAGGACTAGAAGAAAGTCACACATGCCCGATGTACAACCCCCAACGACGTTAGTTGATGTGGGAAATATGGAAACGCTTCACCATATTCTGCATGAATGCAACGAAGCGCGAAGAATACTTCAGGAAATGGGTATACTGGATGAACTAAAAGAGGGAAAGTACACCCAATGGATGCTACTACACAGCAAAAAATTACCGGCGCTGCTGCATACATTGTTTGGCCTTGTCTGGGGGAAGGATGGCGACGCGAGCAGGTGAGATTAGAAAAAAACAACAA**CCTTCATATA

T.congo.pschr.10:2,442,998..2,447,747

ACTTGTCTTTCA**CCCTGGCGCAGCCGGCCACCTCAACGTGGTGCCAGGGTCCAGTACTCTTCATTGGAGAGGAAGCTAAGTGCCAGCTACGCTCTCGATGCTATCGTTTGCGAAGCTGGTTCCTAGCTAAAGGGCCGGAAGGGGGTATATTGAAGCCTAGCTGTAGTCCACCCGTGCGAGCTGTATTGGTCACAACCCTGTCAAATGTCATGAGCGCTCACCTGCGCCATCCCAACCGACAATGTGCCTTGCAAGAAGCACTGCGTCAGAAGTTGCTGTTGTGTGGTGATGTGGAAGCAAATCCTGGACCTCTCACAGTACTTCAACTAAACGTTCAGTCACTGACAAAGACCAAACTTTCTTCCCTGCTGTCCAGGGGGTCGGATATTATTACACTGCAAGAGACGTGGAAATCGGCAAAGAAAATTTTGTCTTTGAAGACATACCCTTAAATTATGTACTCACGTAGAGGACGAGGCGGCGGAGTTGCAATTATGGTAAGGAATACACTGAAGTCAAGACAAGTTACTATGAAAATTCCTGAATATGATACGAACACCGAAGCTGTGCTTGTGGAAGTCATATTACGAAACGGAAGTAATATGTACATCGCTAGTGTCTATCTTCCTCCACCAGCAGTTGTCACGTCAACTTTGCACAAACTTGTGACTACTGTGCCTGCGTCTTCTCCCTTTCTCCTATGTGGTGACTTCAATTTACATCACCCATTGTGGGCATTAAAGGGGGATGAAGCTCCGCCGGATACAGCACAAAAGCTCTTGGACATCACCCTTGATGCGAACCTCTCCCTCGCAAACGAGCCGGGATTTACGTTCGCTAGAGGTCCTACTGAGCGCTCGTGCACTGACCTGACCTTTCAAAGGTTTTTAACTGTTGAATGTTGGACAGCGCGTGTTACCATCCACAGTGATCATTTTTTGATAAAGTTTTCCGTGAGGGCTTCCCACCGCAATGAGATCCCCCCAGCGGCGCCTGTTAGGCGAAGACATTTCTACAGCTGGAAAAAGTGTGACTGGGATTCTTTTCGGAATAAAATGGATTCTCAGCTCCCTAATTTCGATCCTAGAAACATTCATCGGAACATAAAGGCCTTTACGGACTGTTATTATACCGCACTAAGACGACACTTCCCGCGCGGTATGATAAAGGATGGTCCCATTTTCTGGGACCGTGAGATAATAGAAGCTGAACGTTACGTGGAGACACTAAAATCAATATACCTGAATATGCCATCTTCAGCGCAATTGGCGGCGCCGAATGAAGCTAAGGATAAATATATGGACACTGTCAGGCAAAGACTGAACACTACTTTTCGACACCGGCTGGGGAAACTATCCCCTGGAGAGCACCTCTCCTGGAAGTACATCTCGTCACGGAATAGGGCTACCATCCCTCTGCAGACTCCCTTCTTTTAAGTTGTAGGAAACAAGCAGCTTAGTAACTCGCGTCACATTGCGAGCGCATTCAATCGCAAATTCTTCTTCCTATTCAAGATAGGTCAACGTTACTTAAGTTCACTGTCAGAAGATCTTTTAAAAGAGGTGAGAAAAGCTTGTCTCTCTCTTCTGTTGCTCTCTCTTGGTTTTTTCACCTCATTTCTCTTCTCTTCCCTCACTAGGTACACATATAGGTCTTCCTTTTGATGCTGCTTCTTTCCCCACCCTCTCCTCTCTAACTGCACTCTCCTTTCTTGACGCCTCATTCCACCTGGCGGAGCTACTTTCAGCCCTGAGGGATACCCCTTGTGGAAAAGCCCCTGGTCCGGATGGGACTTCTGCCGAAACATTTGGACATTTTTCTGAAAAGACAGTGAAATACCTTTTGAGGTGCATTAACCCAAGCTGATTAACAGGAGTTGTTCCCGTTCAGTGGAGACGCGCAACTGTTATCCCACTACTCAAGCTTGGGAAACGGCCGGATGACACCAACTCGTATAGGTCTATCAGCCTTACATCGGTTATATGTAAGGTAGCTGAAAGAATGGTTCTGAGACGATTATTACGGGTATGGATTCCCCATGCCCACCAGTACGCGTACAGGAAAATGTATACGATGACAATGTAATTGGCGCAATTAGTAGATACTGTTGAGCATAACCGAAATCACTATTTCGATGTCCGCCTCCCTAAAAAGAGCGGCATTGGTGACCAAGTGCACTACAGACCGCATTGCACATTGTTGGTTCTCGTTGATTTCAGTCAAGCTTTTGACTCAATCGACGAACATGTGCTGAGCAAGAGGCTTGCACTTATTCCTGGGGTTTTTTGTAAGAGGTGGCTTCGAAACTTACTATGTGATCGACTTGCACGGACCAGAGTCGGTAATCACAAGAGTGCTCAAAGACCGGTGCTAAGAGGAGTTCCTCAGGGCTCCGTAGTGGGACCATACCTGTTTTCCCTCTACGTGCACCCGCTCCTCAACTTGCCTAATACGGACCCGGAGATATCGGCTGATATCTATGCTGATGACTTATCCATCGCCATAAAGGGCCGATCTCGTGAAGAGGCCGTCCTGCAGGCTGATTCTTATCTGGATAAATTGCATCGATGGACATCAGAAAATGGTCTACAGGTGAACCCGTTAAAGTGTGAAGCCGCGTGGTTTACGATATCAACACACACTGAAGATGATAAGGACCGTGAAGGGAGGTTTCCACTTCTCTTCAATGGTCATGAGATACCCATCTCGACTATGGGATCCACACACCTGCCAAAGCTGCTGGGGGTACCCCTAGACACACGTATGAATTTCAATTCCGCCGCTACTTCTCAATGCGCAGCCACTTCGACCCGAATTGCACAACTGAAAAGTGTGGCGCACAAGAAGGCTGGTCCGCTTCCGCATGACATGCGTACTTTTGTCATTGGTTACGGAGCGTCCAAGCTCTTATATGGAAGTGAGATGATTTGGGCGTTAGCTGACGACTCAGCAAAGAATGCGATGATGAGGACATATGCGAGCCTAGCCAGAATAGTAAGTGGTACATTATCGACGACTGACCCAGTATCTGCGCTACTGGAGGCGAATATGACTCCGTTACACATTCTTGCATTGAGGACGCGCTTTGCTTTATTTGAGCGCGTTCGATCATTCCAAAAAGAATGGATTCGGCGTCCGCCTCCAGAGCCCCCCGCGTAAAGTTTTTCGCATATCACCGATATCGCGAGAGACGATGTATTCACTAGTCGATGATTTAACCGAAGAATATGGAGTCAACCGAAACAGCGTTAGGGAAAGGAGATTCTTCAAGCCTGCTGTTCCTCCGTGGTCAGTTTCCCATGCGAGCAAAGTGGCGTTGGGGCTTACTGTGGAATACGACAAATCTTTCACACACAAAGATGCAATCCGCTCAGCAAAGAAATGGGCCAGTCTACATGAGATTGGTAAGCATAACCACTTTCAGTGGCTGATTGCAACAGATGGTGGTATTCAATCACCCATGCCGGCCGGTGTCAGGCTATTGTTTAAATCCGTCTCGCATCCCGTACTGATGAAGCAAGTCAGCGTGAACTGTGGATCCGTATCAAGCGGTTACAGAGCAGAGTCCGTGGCGATGCTATTGGCTCTAGATAGGTTGGTTATGCCAATGGCGGATGTCAAACATAAGACGTTACTCATTGTCACAGATAGCCAATCCCTCCTGAATGCGTTAAGCAAAGGTCCGCTAAGTCAATGTGACTACACGGAGGATGTGATATGGACTAGACTTATTGAGCTCACACTGCAATGATGGTTAATTCACTTTCAATTCTGTCACAGTCATTGTGGAGTAGTAGTGAATGAGATGGCAGACGAATATGCAACTCAATGCATGGAGAATGGTCACTTCACCGAACCCTCAGTCAAACCACCATGGCATAGAGATCTCGTGGCCCTCATCATCAGACAACTCAAAAAACGGTGGGCCGCCTCATTGAGAACCGACACGCATCGCTACAAGCTGTGTGGAGCGAAACCGTCAGACCTTAGTGGACTGGACCTGATTGATGTTACGAAGTTCACCAGATCAGAAGTGGTTCAGTTGGCTAGATATCATTCTGGATAGTCAGAATATTTCGGACGTTTATTTTGGAGTCTGAGGGACTGCTTGCCGGCATGCAGGTTGTGCAACTGTACGCCAGAACAGGCCGCGGTACTGTCACACTCTTCTCTTCCGGAAGAAGGCCTGGATGCATCCACCAACACAACACAAAAAATGGCGGAGAGGCCAAGGGCAAATAAGAATCGGAGACGAGAACCATGGCCATACTGCGATGCTGTCTTTGTTGGATTTACAAAATTAAAACACACTGTAAAACACAGCATAGTGATCGGCCAAAACCCGCCGAGCAACTTCAATGTGATTTTTGTGGTGAGGAGTACTCCAACAGGAGGAGTACCGCGCAGCACAGAATGAGGTGCAAGCAAAATCCACACTACATTCGGTTAAACAACAGTGGGACCAGAAGAAAGTCACACATGCCGGATGTACACCCCCAACGACGTTAGTTGATGTGGAAAATATGGAAACGCTTCACCACATTCTGCATAGATGCGATGAAGCGCGAAAAACACTTCAGGAAATGGGTATACTGGATGAACTAAAGGAAGGAAAGTACACCCAATGGATGCTACTACACAGCAAAAAATTACTGGCGCTGCTGCATACATTGTTTGGCGTTGTTTGGGGGGAGGAGGAGGATGGCGGCGCGCGCAGGTGAGATTAGAAAAAAAAAAAA**CTTGTCCTTCC

*Trypanosoma congolense* NAR*Tco* sequences:

T.congo.pschr.1:434,515..435,423

TATTTATGACTTGACGCGAC**CCCTGGCGCAGCCGGCCACCTCAACGTGGTGCCAGGGTCCAGTACTCTTCATTGGAGAGGAAGCAAAGTGCCAGCTACGCTCTCGATGCTATCGTGTGCGAAGCTGGTTCCTAGCTAAAGGGCCGGAAGGGTGTATATTGAAGCCTAGCTGTAGGCCACCCGTGCGAGTTGTATTATCTACAACTGTGTCAAATGTCATGAGCGCTCACCTGCGCCATCCCAACCGACAATGTGCCTTGCAAGAAGCACTGCGTCAGAAGTTGCTGTTGTGTGGTGATGTGGAAGCAAATCCTGGACCTCTCACAGTACTTCAACTAAACGTTCAGTCACTGACAAAGACTAAACTTTCTTCCCTGCTGTCCAGGGGGTCGGATATTATTATAGTGCAAGAGACTGGAAATCGGAAAAGGATTTCTTGCCTTGAATACATATCCTTAAATTATGTACCCTTACCACCCTAAAGAAAAAGGCGCGGAATTTCAATTATGGTAGGAAAACCTGAAATTCAAAAAAGTTTATATGAAAATTCCGGAAAAAAAACAAACCCGAAGCTGTCTTTGTGGAATCCGGCTAATAATAGTGGGACCAGAAGAAAGTCGCACATGCCCGATGTACAACCCCCAACGACGTTAGTTGATGTGGGAAATATGGAAACGCTTCACCACATTCTGCACGAATGCAACGAAGCGCGAAGAATACTTCAGGAAATGGGTATACTCGATGAACTAAAAGAAGGAAAGTATACCCAATGGATGCTACTACACAGCAAAAAATTACTGACGCTGCTGCATACATTGTTTGGCCTTGTCTGGGAGGAGGATGGCGACGCGCGCAGGTGAGATTAGAAAAAAAAAAAAAAAAAAAAAAAA**CTTGACGCGACGCAACCTGG

*Trypanosoma vivax Ingi* sequences:

TvY486_10:3,169,044..3,173,845

AAGTAGGTTTTC**CCCTGTTGACGCCGCCCGCCCCACCGTCGTGCCAGGGCCTGGCGCTCCGCCTGGGAGGAAGCCGAGCGCCCGCACCATGCCCGGTCCCACAGGATTGGGCGGGCAAGAGGCTGACGGCAACCAGAGAGGAAGTATGCAGCACCACCGGCACTCTGGGGTCAATGAAGTTGTTCGAATACTTCCCCGCACGTGCGGGCGTGCTACGTTAGATGCGAGGCGGCTCCTGCTGCTTATCGACGGAGACGTTGAGCGCATCCCTGGTCCTCTGATGCGTGGAGCCCAGTGGAACTCTGGGGGTCTCTCCCAGGCGAAGCGGGTTGCCCTGGAGAGGAAGCTCCGTGAGGACATGGTTTTGTTTTGTCCCTTGCAGGAGGCGCGCCTGGCGTCGGCGGAGTGTGCCGCGCTAAAAATAGGCGGATGCCAGCACGTGGGCCAGGCGAGGACGCCTCACGGGGATGGGGTGTCGATTTTGGTTAGGGACAGAGTGGGTGTAGAGGTGGGCGTTCTAGACGAAAAGGTTCCGGAGAGAGCGGCAGTGACACTGAGGTTCTCAGCCAACGTGAGTCTAACGATCAAAACGGCAGACTTCCAGAAGAAGGACAGACGTTTCCAGCGAGTCGCTTGACACCTTGCTGGGAGCAAGCGGGCCATTGGCAGTAGGAGCGGACATGTGCTCACACCACGTGTTGTGGTATCCGTTTCGCCCGAGTGACGACAAGAGAGAGTGCATAGCCGACTGGTGCGCGAAGAACGGCCTGTCGATTGCCAATGCCGGGTCGGCTACCAGGCGACAGTCGGGCACGGCAGCACTTTCGTCACCGGACATCGCGCTTTGCAGAGGCAGTGGAATTTCCAACCGGAAGTCCGCGCTCAGACCGGACAGTGACCACCATTGGATCACGTTCGATGCGTTCGCGGGCACCGGCCTGAACGCGATTGCTCCCTCCAAACCCGCCCGTGCACCGTACGCGTGGAACAAGGCGAGGTGGAACGAGTTTAGAAAACTGAGTGACGAGTTTATATATCCGAAGAATGAAGAGGTCGGCTAAGGGCGCGGATGCCATGAACGAGGCGGTGGCGAGGGCCATCCGGATGGCCGCTAGGAGGACAGTCCCCAAGGGCAAGGGCGTGGCGCTGCCGTTTTGGACGCCGGAGCCGGCGAAGCTGAACAGAATGGTTCAGGAGCGCAAGAACGAACGGAAGATGAATGCGCCGATCCGCTGGCGGAGGAAGGTGCTTGCTGACACGGCGTTGGGTCGGTGGAAGGAGAATGTGCCGAAGCTGTCGGCCACGGATTTGGCGAGCTGGAACCTGGCGAAGTCGATATATGCGCCGCGGCCGCTGACGTCGCCGGTGCTGGTGGTGGATGGCCATCCGCTGACCAAGCACCACCAGGCGCAGGCATTGCCCAAATGCACATGGTCAAGTCAACGAAGGCACCGCGTGCACCAGAAATGAAGATACCGAGCACCAGGCGAGGCACATTCCAACCCATCACCGAGGCAGAGCTGGATGTCGTGCTGTGCGAGCTGTCTTCCGGCACGGCGCCGGTTGATGATGAGATCCACTGTGAGGGGCTGGGCCAGCTTGGCAGGGGGTCAAGAAGGTGCATTTTGCGTCTGTTCAACTACAGCTTGCGTGCGGGGCAGGTGCCAGCCAAGTGGAGGCATGGCACCATAGTCCCGCTGCTGAAGCCAAACAAGCCAGCGAACAGCGTGGCGTCTTTTCGGCCGGCGACGCTTACGAGCGCGCTGTGCAAGCTAATGGAACGCATCGCGGCGCGCCGCGTTAGGGATTGCATCGAGGACAAACTACAGCCAGAGCAGGCATGGTTCAGGCCGGCAAGATCGACGCTTGGCACGCTCATGCAGATGACGAGTGCAGTGCGGCGAAGGAAGGATGGGGAGAAGACGGAGCCTGTGTTCATTGACTATGCGCGCGCCTTTGATTCCGTGGATCACGGTTGCATTGTCAAGGAGCTGCTGTCCTTTGGCGTGGAAAAACATCTGGTGGCGTGGATCGCTGGCTTCCTGAAGGAGCGCACGGCGCAGGTGCGGGTGAACAACGTGCTGCCGGAGGAAATCAGCCCCAGCTGTGGCGTCCCTCAGGGCTCGGTGCTGGGACCGCTGCTGTTCATTGTCACGGCGGATTCGCTGAGCAAGCGGCTCAACTGCATCCCTGGGCTGAAGCATGGGTTCTTCACACGCGACCTTGCAATTGCGTGAACAAGCGCTGGCCTAAGCGAAATCCAGCAGACCATCCAGCAGGGATTGGACCGCATCACGAACTGGTCGGCAGAGTGCTACATGGAGGTGTCTGCGGCGAAGACTGAGCACACGCTATTCGGTGCGCGGAAAACGAGCCTACTGAGCCTGAAGGTTGGAGAGACTGTGCTGAAGGAAGCTCGCGCTCCGAGGCTGTTCGGTCTCACCATGCAGCCGAACAAGGGGCTGAACAAGCATGCGCTGAGAATGAGTGCAGCGGCCGGCTCGCAGCTCACGCAATTCAGTGCAGTGGCGTCGCCTGAGTGGTGTCCGGATAGGGAGAAGTTGCGCGCCTTTTACCTTGTACTGGTACAGGCCAAGATGTGCTATGGCGTCGCGTCGTGGCGGTTCGATACTTCGCTGTTGGATCGCGAGCGGCTGGAGAGGGTGCAGGCACAGGCGGCACACATAGTTGCGGGTATTCCCAAGGCTGCCAATCGTGAAGATGCCCTGCGTGAGGCGCGGTTGAAACCGATCAACGAGGTGGCACACCGGAGGGCGTTGGAATATTACCTGCGATTGAAGGTCAAAGGTCCAGTGCATGCGAAGGTGGGGGACAGCATCTTCCCGCCCGAACACCCAATCCACGTCAGGCTTGCGAAGGTACAGCACTTGTGCAGCATCATTGATAGCCTCGAAAAACCGCACGACGCGAAGGTGTTGCAGCTGCTCAGGCGGATTCGCTTCAACATCGCCACGCCGGGCGGCCTCAAGGCGGACGCACCAGAGAAGGACAAGAAGATGCACACCATGCGGCGCGTGCAGCGGTTCAGCGACTTTGACTATCAGGTGTGGACGGACAGGTCGGTGGTGCTGGATGTCTCGTCATGAGCCGGAGCGCTGGTGTACCCGAAGGATGGTCGGCGTGAGAAGGTGGTGCTGGAAGCTGGGTCGCTTGTCTGCAGTTACCGTGCGGAATGTGTGGCGATGGAAGCAGGCTTGAAGAGGCTCGTGGATGTCATTGAGCTGAGCAAGACACACAGGACGCGGGTGGTGGCATTCACAGACTCACTGTTGCTGTTGATGCTGGGCGCTGGTCCTGCAGGGGTGGACGGCGCGATGCTGAGGCGCATTTGGGATCTTATCCTGCACATTGTGCGGCTCCGCGCGTCCGTCAACTTTCAGTTCGTGTTCTCGCACTGTGGGGTCCCACGCAACGCGGGGGCAGACAAGGCAGCTGAGCAGGGGAACGTAAAGCCGCAGTCGCGTCCGGCGTGAATCGCTGACATCGTCACTGGTGTAGAGAGGCAGGTGCGGAACGAGATATACAGGGCCTTTGAGGATGGTCGGATGCCACGGACGCATCGCAGTGTGCTACTCGATCACGTTCGCCCAGCGCCGAAGCACTCCAAGCTGGATTATTGCGAGTCGTTATTGGCGCAGTTCAGAACAGGCACATCGGAGCATTTCGGGTGGCCACGCAGAGTGCTCACACGTAAGACGGACCAGCTAGAGTGCAGATGGCGCAGCACGCAGCGCGCTGGGAGTGATGCAGCACAGGAACACCCCTCGGCGGAGCAAGTAACGGACAGTGAGACTGCACCCGACCTTGGGATAGCGACCAGGCAGGGCGACCCGATTACCTGCCTGTTGTGCAACATGGTTTGCTCGTGTCGGCAGGCAGGTGTAGTGCACCTAGTAAAGATTCATGGTCTGGAGAGGGATTGCGCATTGGCACTGGCCAAGAAAGCCAGGCGTGCAGCGCTGACGTACAAGAATGGATACACCTACCATGTTTGTGGCTATGTCTTTGAGCGGCGGGGACTACTCGTGGAGCACAAGGCACAGCACCCTCCGGATGTAGTGCCAATCGTTGAGGAACGTTCAAAAAGGCCAAGGGAAGAAGACGCGACCGACGATGGCAACGCGCTCAAGTGCCCCTGGTGTGCGAAGAAGTGGGCCGGACACGCGTGGCTGAGGAAGCATATGGTGAAGAAACACGCGGAAAAGCAGCTGTGGAGTGGCACCACGGAGGCTGAGGACACACCCAACAGCGATGACGAGGCAAAGCAGGAGGAACATGAGCAGACGGAATTTGTATGCCAGCAGTGCCATCGCGTCCTCAAGAGCAAGACGTGGCTCACCAGGCACAAGTGCGAACCCACCTCTATCATAAAGTCGGAAGGCTCGAACGTGGCGGAGCAGCCGGTCACAGCAGCGTGTCCCATTTGCGGCAAGGAGTACCATTACAGATGGCTGCTGCGGCACATGCTGGCGAAGCATCGCGCCACAACGAGTCATTACGTCCTCAGCCGCGCACAAAGCCCAAGCGAAAGGAGATGAGGACAGAGGCTCAGGCACAGGGCGAGGGGAGTGGGCCACTGGAGTCATTTTGGGGAAAGGGCGGAAACATCCTCTCGGGGGTGGTTCTCCAGTAAACGCGATCTTCCTCAAGTTTCCCCTTTGGTCGACGAGGGAGGAAATTATGTCCAGTTCAGCAGTCCATATCAATGTGCTCGATTGTAGAAGCGATTGCGAACCGAGCGAGTGACAATACAACGAAAACCAAGAAAAAAA**GATAGTTTC

Note that the same locus is shared by LmjF.29:1010562..1011142; LinJ.29:1,017,989..1,018,568; LtaP29:986,367..986,974; and Lmex.08_29:190,165..190,764 in the different *Leishmania spp.* The first three were successfully screened as described in Methods, but there were no significant hit in *Leishamina mexicana* neither *L. braziliensis*. We blasted the homologous intergenic region locus in *L. mexicana* and *L. braziliensis* with LmjF.29:1010562..1011142 as query and significant results were obtained in the first one (Lmex.08_29:190,165..190,764) but not in the second one. The surrounding region of the insertion is missed in a gap in the genome annotation of *L. brazilienzis* genome, so we can not conclude if the insertion occurred before or after *L. brazilienzis* divergence.


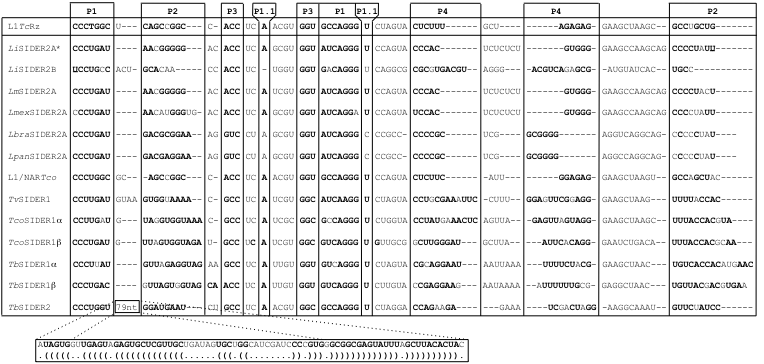


**Supplementary table 1. Selected ribozymes for the present study.** The table shows a sequence alignment of the different ribozymes shown in figure 2. In the top of the table are shown the corresponding helixes and pseudoknots of the manual ribozyme folding according to the previously proposed folding for L1*Tc*Rz (first line, [1]). Bold regions are predicted to base pairs with the corresponding region of the same helix. *Li*SIDER2A, *Lm*SIDER2A, *Lmex*SIDER2A, *Lbra*SIDER2A and *Lpan*SIDER2A sequences are those corresponding to the insertion 1 referred in the text. *Note that *Li*SIDER2A and *Ld*SIDER2A have identical sequences of the ribozyme encoded in the signature I and in the upstream 20 nt.


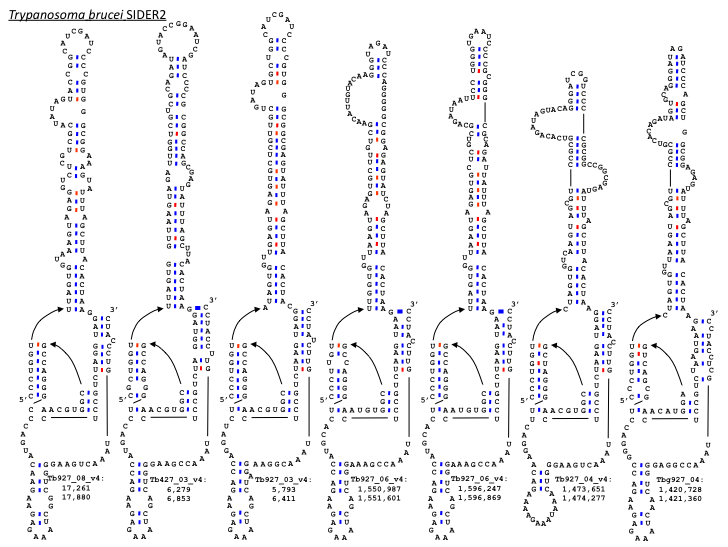


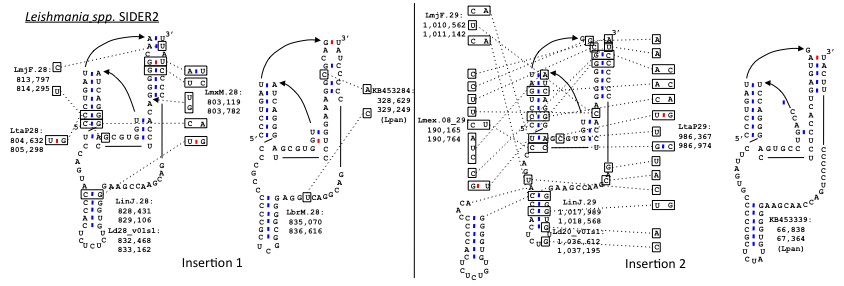


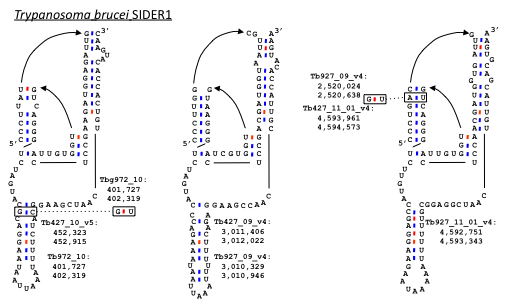

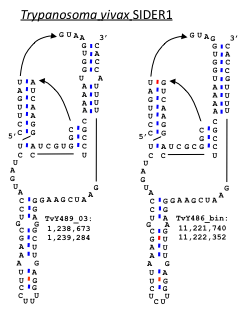


continue in the next page


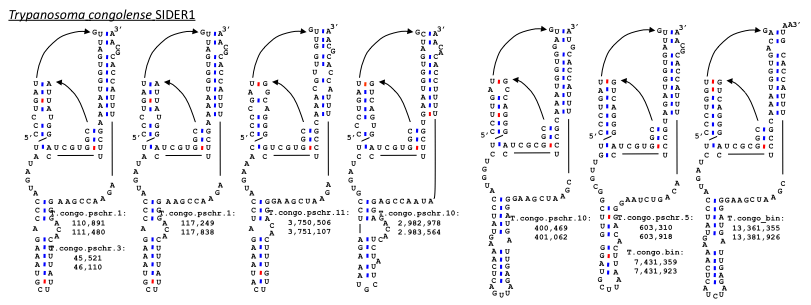


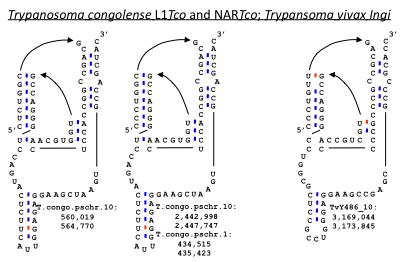


**Supplementary figure 1. Manual folding of the Pr77 signatures underlined in the sequences depicted above.** Watson-Crick base pairs are depicted in blue and wobble base pairs in red. Dotted-lines connected to external boxes represent nucleotide changes in the corresponding sequences respect to the shown folding.


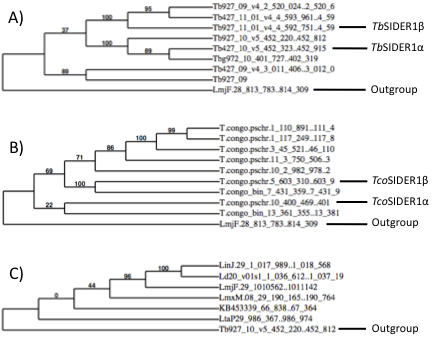


**Supplementary figure 2. Phylogenetic analysis of *Tco*SIDER1 and *Tb*SIDER1.** The figure shows a cladogram obtained for the different full-length *Tb*SIDER1 (A), *Tco*SIDER1 (B) sequences and insertion 2 *Leishmania spp.* SIDER2 (C). The selected ribozymes for the present study are indicated as *Tb*SIDER1α, *Tb*SIDER1β, *Tco*SIDER1α and *Tco*SIDER1β. Branch support values are shown in %.


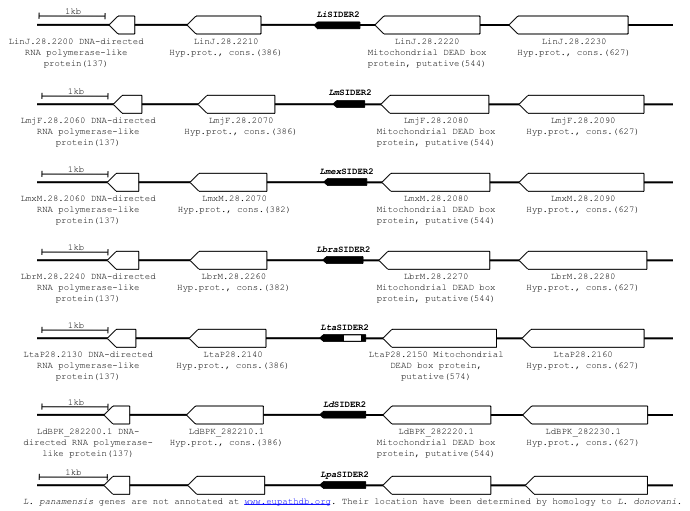


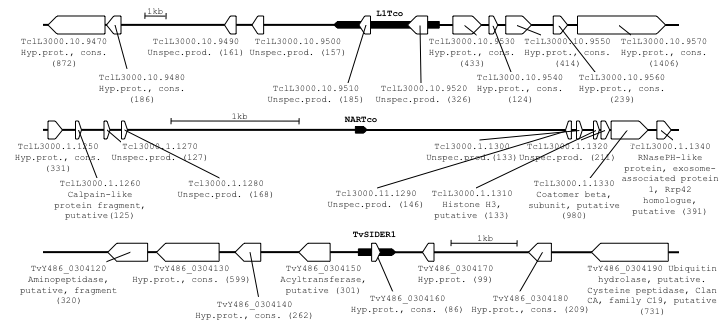


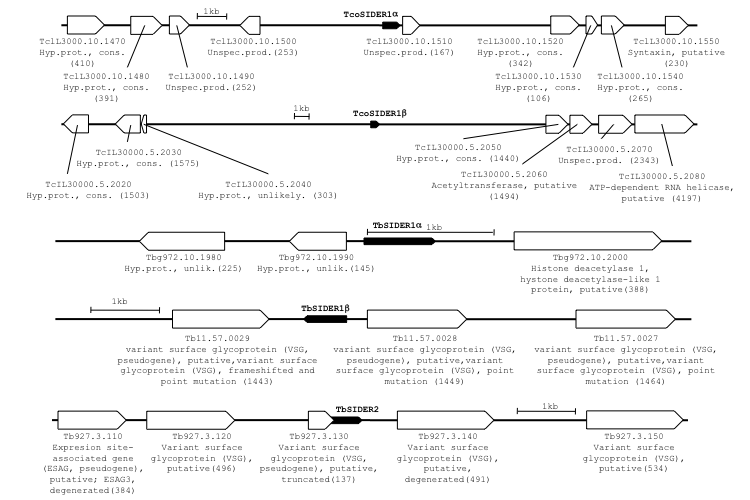


**Supplementary figure 3.** Detailed genomic position of the selected mobile elements.


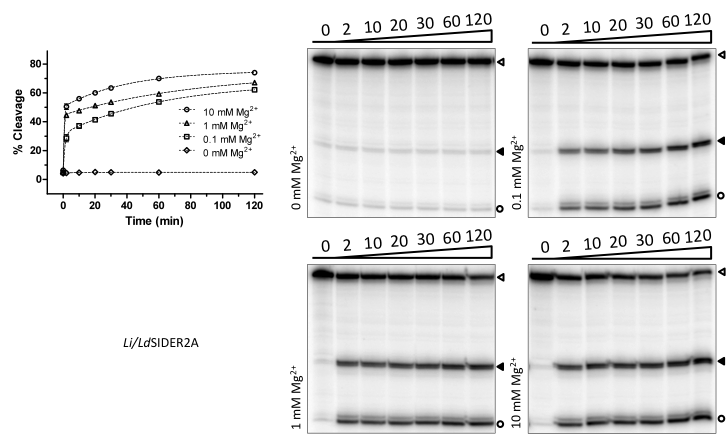


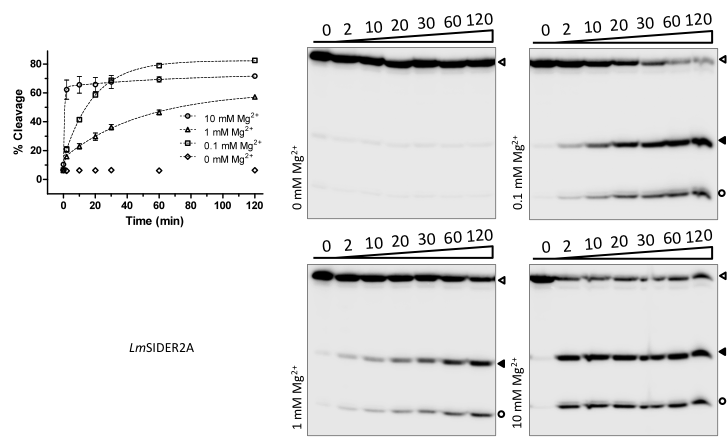


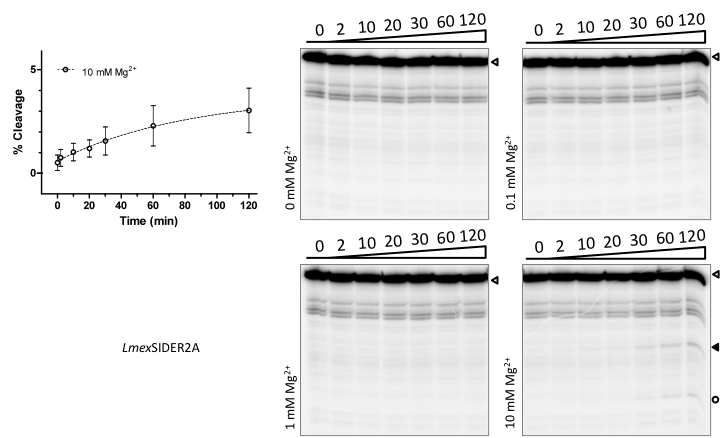


continue in the next page


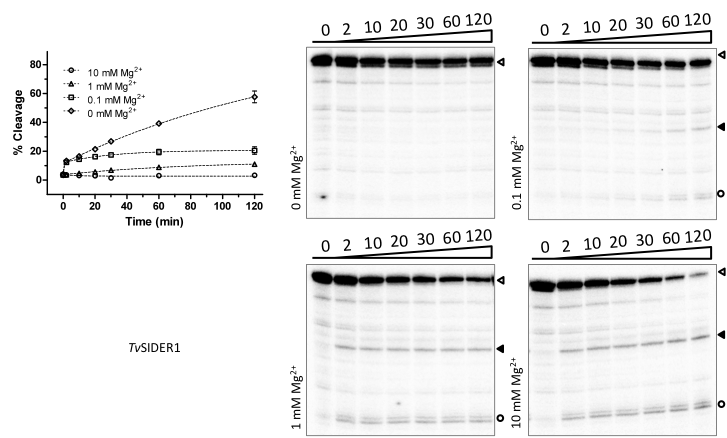


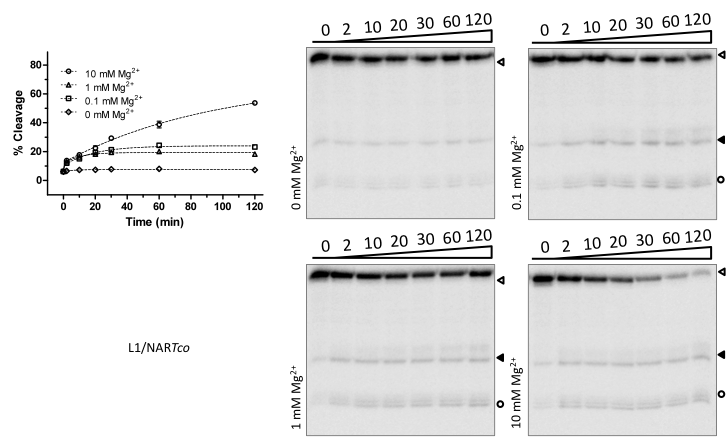


**Supplementary figure 4. HDV-like ribozyme kinetics.** In each panel it is shown a time course ribozyme kinetic at a different magnesium concentration (depicted in the left-bottom corner). Time (min) of each line is shown above. The quantification of triplicates of each kinetic is plotted on the left of each ribozyme analysis. Data fitted to a two-phase exponential decay model. White arrowheads indicate the uncleaved fragment; black arrowheads indicate the cleavage 3’-fragment; and white circles indicate the cleavage 5’-fragment.


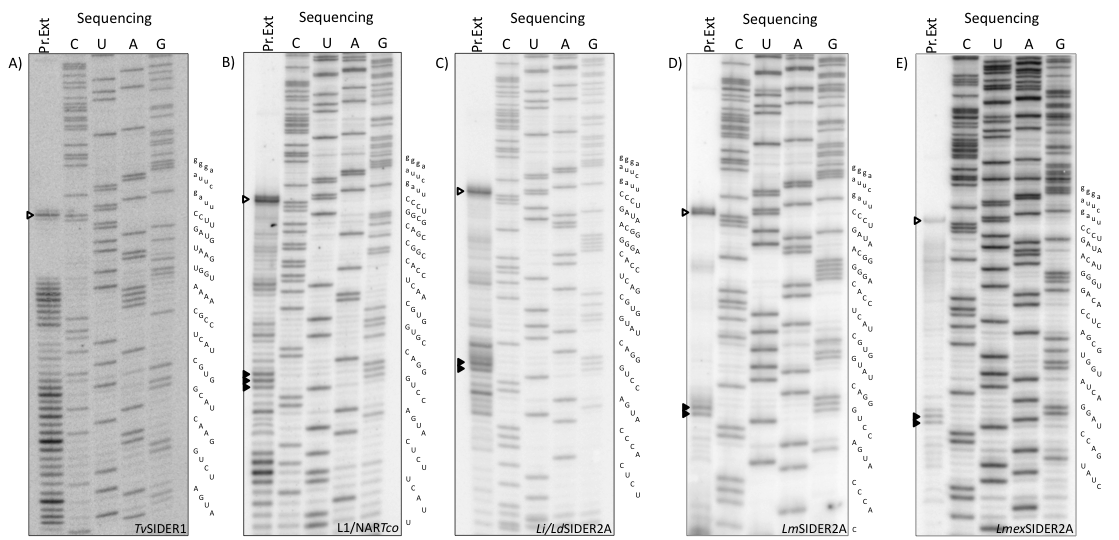


**Supplementary figure 5. Determination of HDV-like ribozymes cleavage point by primer extension.** Each gel shows the result of a primer extension reaction using the cleavage 3’-fragment of the co-transcriptional cleavage of each ribozyme as template and a reverse primer specific for each ribozyme. The same primer was used for a sequencing reaction using each ribozyme DNA construct as template. The white arrowhead points the maximum extension product and the black arrowheads points the helix P1 internal stop of the extension.


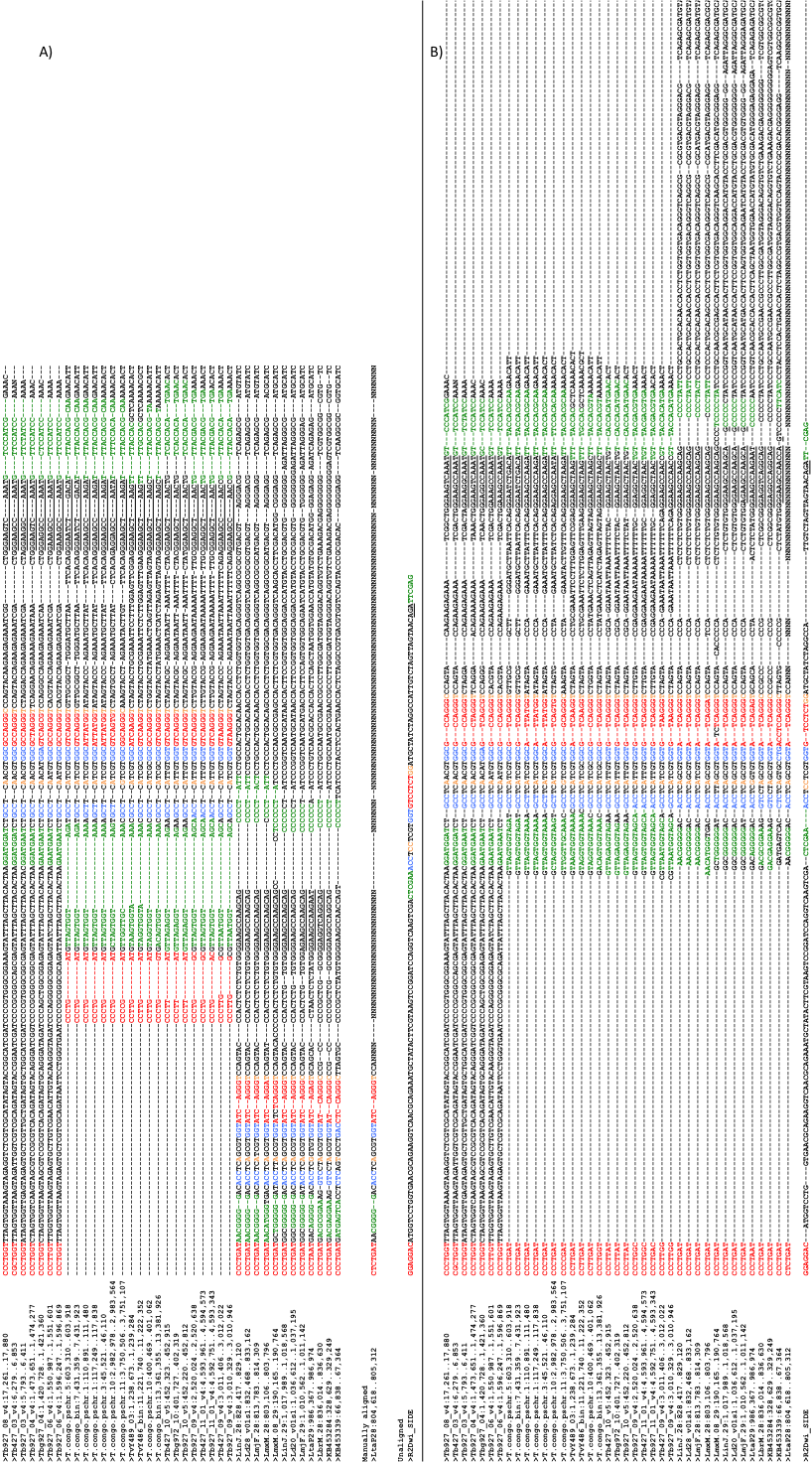


**Supplementary figure 6. Manually corrected *in silico* alignments of SIDER Pr77 signatures.** SIDER Pr77 signatures were *in silico* aligned excluding R2Dwi_SIDE and LtaP28:804,618..805,312 sequences to prevent perturbations derived from the high divergence of the existence of a track of unknown nucleotides respectively (A). Manual re-alignment of the structural region of the ribozymes according to manual folding depicted in supplementary figure 1 (B). Color code: red, helix P1; green, helix P2; blue, pseudoknot P3; orange, pseudoknot P1.1; underlined, junction P4-P2.


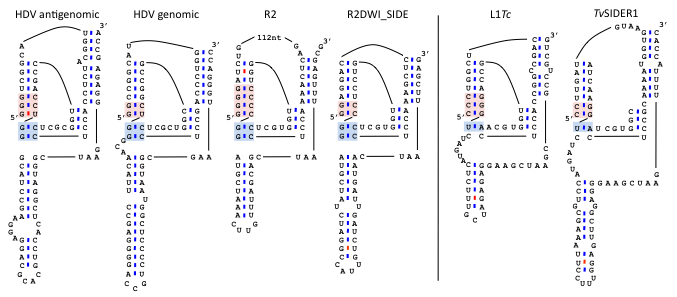


**Supplementary figure 7. SIDERs full-length evolution and trypanosomatids ribozyme differences compared to previously described HDV-like ribozymes.** The secondary structure prediction of HDV antigenomic and genomic ribozyme [2], R2 ribozyme [3], R2Dwi_SIDE [4], L1*Tc*Rz [1] and here described *Tv*SIDER1Rz.

**REFERENCES**

1. Sanchez-Luque FJ, Lopez MC, Macias F, Alonso C, Thomas MC: **Identification of an hepatitis delta virus-like ribozyme at the mRNA 5'-end of the L1Tc retrotransposon from Trypanosoma cruzi.** *Nucleic Acids Res* 2011, **39**:8065-8077.

2. Ferre-D'Amare AR, Zhou K, Doudna JA: **Crystal structure of a hepatitis delta virus ribozyme.** *Nature* 1998, **395**:567-574.

3. Eickbush DG, Eickbush TH: **R2 retrotransposons encode a self-cleaving ribozyme for processing from an rRNA cotranscript.** *Mol Cell Biol* 2010, **30**:3142-3150.

4. Eickbush DG, Eickbush TH: **R2 and R2/R1 hybrid non-autonomous retrotransposons derived by internal deletions of full-length elements.** *Mob DNA* 2012, **3**:10.
